# Enhancement of rime algorithm using quadratic interpolation learning for parameters identification of photovoltaic models

**DOI:** 10.1038/s41598-025-04589-x

**Published:** 2025-07-01

**Authors:** Shazly A. Mohamed, Abdullah M. Shaheen, Mohammed H. Alqahtani, Badr M. Al Faiya

**Affiliations:** 1 Electrical Engineering Department, Faculty of Engineering, South Valley University, Qena, 83523 Egypt; 2 Department of Electrical Engineering, Faculty of Engineering, Suez University, P.O. Box: 43221, Suez, Egypt; 3 Department of Electrical Engineering, College of Engineering, Prince Sattam bin Abdulaziz University, Al Kharj, 16278 Saudi Arabia; 4 Department of Electrical and Electronics Engineering, Faculty of Engineering and Computer Science, Jazan University, Jizan, 45142 Saudi Arabia

**Keywords:** Rime Physics-based optimization algorithm, Quadratic interpolation learning strategy, PV parameters extraction, Practical solar modules, Electrical and electronic engineering, Solar energy

## Abstract

Accurate parameter estimation in photovoltaic (PV) models is essential for optimizing solar energy systems, enhancing their efficiency, and ensuring precise performance predictions. This paper proposes a novel Improved version of Rime Metaheuristic Optimization (RMO) influenced by rime growth and combined with Quadratic Interpolation Learning (QIL) technique for the simulation and design of the triple-Diode Model (DM). This novel combination seeks to provide a more accurate perspective in the field of solar energy optimization by managing the complexities of PV module characterization with greater flexibility and resilience. By meticulously replicating the distinctive features of both processes, the hard-rime puncture and soft-rime searching are disclosed. The QIL technique improves the search process by selecting three different rime particles rather than relying solely on the current best solution. This selection allows for a more diverse set of candidate solutions, fostering better exploration and reducing premature convergence to local optima. By leveraging quadratic interpolation, QIL adjusts the solution updates in a flexible and nonlinear manner, enabling a more precise and adaptive parameter estimation process. QIL’s capacity to adjust its quadratic function in a flexible and non-linear way makes it easier to navigate complex terrain. The novel IRMO as well as the original RMO are developed for predicting PV parameters for the triple-diode model (DM) of the three distinct PV modules which are Photowatt PWP201, STM6-40/36, and R.T.C France. In accordance with other published publications, the results of the suggested IRMO are also compared with those of contemporary algorithms. According to the results of the simulation, the upgraded IRMO shows significant average improvements of 49.56%, 62.56%, and 34.15% for the three modules, correspondingly.

**Supplementary Information:**

The online version contains supplementary material available at 10.1038/s41598-025-04589-x.

## Introduction

In the twenty-first century, there have been constant changes to solar energy-based electricity production. Enhanced field studies and the unlimited supply of sunlight during the day in many African and other nations are credited with the rise in solar-generated electricity^1,2^. As a result of that, field research is crucial for precise cell and PV module structure design that anticipates the PV system’s generation of electricity^3,4^. PV modelling considers well-known characteristics such series resistance, shunt resistance, the diode ideality factor, photocurrent production, and current saturation^5^. The mathematical representation and visual PV cell corresponding circuit allow one to calculate provide maximum power, undetermined parameters, and comprehend information from a basic technical data sheet^6^.

### Motivation of the study

Various methods (such as numerical, analytical, metaheuristic, and hybrid approaches) have been employed in the literature to extract distinct parameters. The optimum approach is thought to be a metaheuristic algorithm^7^. To determine the unidentified parameters of the PV cell, a collection of nonlinear formulas has been employed in PV modelling. Three distinct approaches, which are analytical, numerical, and progressive computational techniques, can be used to model PV equations, according to a comprehensive assessment of the literature on extraction strategies summarized in^8^.

The most common approach yields precise results with minimal calculation time. Analytical methods are fairly straightforward, and frequently the only thing that is needed to get the desired outcome is one iteration. Ref^9^ has provided a developed approximation approach that functions as an explicit model capitalizing for VI attributes and a determination of the single PV model of diode attributes. Other techniques have been discussed in the literature, including curve-fitting techniques^10^, the extension of the Taylor series^11^, Lambert’s W-function^12^, and a set of approximation explicit equations^13^. While some techniques extract only shunt and series resistances, others allow estimation of five parameters. This analytical method’s main flaw is that it only works with standard test settings; however, under other conditions, it collapses^14^. Curve fitting techniques combined with numerical methods are superior to analytical methods in terms of accuracy of results. The Levenberg technique is used to assess all PV-IV curve points and obtain solar cell characteristics. Although the Newton-Raphson approach is an effective way to delay convergence, it fails for faulty initial guess convergence because it requires huge processing times and storage^15^.

### Related works

The estimation of PV parameters involved the optimization of constraints through the application of metaheuristics. Algorithms mostly draw inspiration from natural processes found in the environment. These algorithms have been categorized as the best since they produce the best outcomes under all circumstances. Curve fitting techniques combined with numerical methods are superior to analytical methods in terms of the accuracy of results. The Levenberg technique is used to assess all PV-IV curve points and obtain solar cell characteristics^16^. Although the Newton-Raphson approach is an effective way to delay convergence, it fails for faulty initial guess convergence because it requires huge processing times and storage. The estimation of PV parameters involves the optimization of constraints through the application of metaheuristics. Algorithms mostly draw inspiration from natural processes found in the environment. These algorithms have been categorized as the best since they produce the best outcomes under all circumstances. Many algorithms that are available in the literature illustrate various modeling approaches, including fundamental progressive computing and traditional GA approaches.

In^17^, Particle Swarm Optimization (PSO) has been applied on and applied on the one-DM and two-DM of solar PV cells with significant implementation but it has been stuck in local minima. Self-adaptive ensemble has been combined with differential evolution in^18^ and applied on the one-DM and two-DM of PhotoWatt-PWP201 and RTC France Silicon cell. The combined technique has been developed with high robustness and accuracy, however, it suffered from high computation time. Orthogonally Adapted Harris Hawks Technique (OAHHT) has been elaborated in^19^ and applied on the one-DM and two-DM of PVM 752 GaAs and SM55 with high convergence rates. Performance-Guided JAYA (PGJAYA) has been illustrated in^20^ and employed on the one-DM and two-DM of PhotoWatt-PWP201 and RTC France Silicon cell with acceptable accuracy but it has insufficient reliability. Marine Predators Algorithm (MPA) has been manifested in^21^ and applied on the two-DM of MSX-60 and KC200GT. The convergence of this technique has first occurred in the procedure. Also, a stochastic Slime Mould Algorithm (SMA) has been manifested in^22^ on the one-DM and two-DM of PhotoWatt-PWP201 and RTC France Silicon cell with substantial exploitation and exploration, however, it requires long time to compute. A method for estimating PV parameters called Multi-Strategy Gradient-Based Optimisation (MSGBO) was presented in^23^. This study changed the Gradient Search Rule based on the quasi-Newton approach by dynamically altering vector motion, and it also devised a crossover mechanism and a Novel Refresh Operator. The shown MAGBO was verified using many PV models, such as STM6-40/36, Photowatt-PWP, and PVM 752, as well as CEC2021 benchmark functions. The comparisons that were put into practice performed better than several coded metaheuristics. For its wider use in further studies, it will be essential to solve its constraints, which include scalability, dynamic model adaptation, and computing efficiency in multi-objective scenarios. Additionally, only one and two-DM applications were offered. By modelling the synthesis and stabilisation of chemical compounds and imitating the behaviour of ionic and covalent bonding in^24^, the Material Generation Algorithm (MGA) was created for PV parameter estimation in order to provide new candidate solutions. The RTC France and Kyocera KC200GT PV modules’ specifications were estimated using the MGA. Considering one and two-DMs, the MGA optimiser continuously outperforms other algorithms in lowering error under a variety of weather circumstances. However, more improvements were needed to ensure the MGA’s stability.

In^26^, Rao technique has been characterized and applied on the one-DM and two-DM of PhotoWatt-PWP201 and RTC France Si cell with high ability to explore, however, the tests for the modules have not been conducted. In^27^, Improved Mouth-Flame Optimization (IMFO) has been signified and employed on the one-DM and two-DM of CS6P-240P module. Moreover, Shuffled Frog Leaping Algorithm (SFLA)^28^ has been applied on the one-DM of KC200GT and MSX-60 with excellent convergence but it has lots of control parameters and the solution was not accurate. In^29^, Improved JAYA (IJAYA), which is a simpler and effective technique, has been demonstrated on the one-DM and two-DM of RTC France Silicon cell but it showcased outcomes with low accuracy. A Hunter-Prey-Based Optimization has been manifested in^32^ and employed on the one-DM, two-DM, triple-DM of STM6-40/36 module and RTC France cell with reasonable degree of accuracy. With significant changes to increase the effectiveness of searches in circuit model parameter estimation, an Improved variant of the Brown-Bear Technique (IBBT) was presented^25^. Fractional-order Chaos maps (FC maps), that allow adaptable adjustment of the algorithm’s parameters for improved exploration, are one of the improvements. Furthermore, by employing the Hippopotamus Optimizer algorithm’s processes, IBBT improves exploitation capabilities by using contextual information to update positions more efficiently. These changes result in more accurate and effective parameter optimisation by better balancing local and global search. However, the complexity of the applications was limited to only one and two-DMs. An Improved Heap-Based Algorithm (IHBA) has been characterized in^33^ and applied on the one-DM, two-DM, triple-DM of SQ_150 PV module and PVM 752 GaAs. The robustness and convergence of this technique are high; however, it requires a long time to compute. The artificial bee colony and the teaching-learning-based optimizer were combined in^34^. This algorithm was used to determine the one-DM and two-DM ‘s unknown parameters. Its efficacy was further demonstrated by comparison with several competing algorithms. Additionally, a heap-based optimizer was established to look for three PV models’ unknown parameters^35^. For the purpose of switching among creating a random individual throughout the search space and modifying the present location in line with the enhancing core of the SMA, chaotic maps were used in^36^ as an alternative to the randomly produced number. The authors argue that SMA has a more useful exploration pattern for the reason to the chaotic maps. Furthermore, SMA was enhanced with the Nelder-Mead simplex technique to accelerate up convergence and produce superior outcomes with reduced function trials. The aforementioned version of SMA, which was used to determine the undetermined parameters of PV models, was commonly known as CCNMSMA. Two effective approaches were applied to improve the generalized typical distribution optimizer, resulting in the creation of an entirely novel optimizer called IGNDO^37^. To estimate the TDM’s unknown parameters, this optimizer was employed. It achieved impressive results when compared to the outcomes of several other computational algorithms. On the other hand, this method requires a little greater computing cost. Furthermore, it still suffers from a slow convergence speed issue given that it requires multiple function assessments to converge on the necessary findings.

### Problem statement

The adequate estimation of PV parameters is a crucial challenge in solar energy optimization, as it directly impacts system performance and efficiency. Analytical methods, such as curve-fitting techniques and explicit mathematical formulations, provide fast solutions but lack generalizability beyond standard test conditions. Numerical techniques, including Newton-Raphson and Levenberg-Marquardt, improve accuracy but suffer from high computational cost and convergence issues, particularly when initial conditions are not well-defined. Metaheuristic algorithms have demonstrated superior performance in handling the nonlinear and multimodal nature of PV parameter estimation. However, existing metaheuristic-based solutions still face challenges such as premature convergence, insufficient exploration-exploitation balance, and computational inefficiencies. Therefore, this study introduces an Improved Rime Metaheuristic Optimization (IRMO) algorithm, which integrates the Quadratic Interpolation Learning (QIL) strategy to enhance solution diversity and exploration capabilities.

### Paper contributions

H. Su et al. have just released the Rime-inspired Metaheuristic Optimization (RMO) method^39^. Through encouraging the rime particles’ evolution into soft and hard forms. To imitate circumstances in the environment, rime particles go through the soft-rime Search Process (SP) and the hard-rime Puncture Process (PP). The SP mimics the gradual accumulation of soft-rime under moderate wind and temperature conditions, leading to smooth, feather-like ice formations. In the context of optimization, SP serves as an exploratory search mechanism, allowing solutions to move toward promising areas with a degree of randomness. Conversely, the PP represents the formation of dense, frost-like layers under stronger wind conditions, resulting in a more rigid ice structure. This process is translated into optimization as a local refinement strategy, where solutions undergo more directed movements toward high-quality candidates.

Extensive research has highlighted RIME’s simplicity, adaptability, and computational efficiency, making it a versatile tool for addressing diverse optimization challenges. As a result, it has been successfully applied in various fields, including path planning of unmanned surface vehicles^40^, lane detection^41^, fault diagnosis of rolling bearings^42^, and engineering optimization^43^. Although a number of RIME modifications have been put out to increase the algorithm’s efficiency in global searches, the algorithm is still in the early stages and needs more work to become more robust. Furthermore, despite its promise, little research has been done on using RIME in solar energy estimation, suggesting a direction for further study and advancement. In this paper, a Quadratic Interpolation Learning (QIL) approach is integrated with an Improved RMO (IRMO). The proposed integration of the QIL approach with the provided IRMO method provides diversity to the solutions by combining information gathered from three separate rime agents rather than depending just on the best one. The donated randomisation and variations offer powerful searching for exploring space. The suggested IRMO approach is intended to detect the characteristics of PV modules by considering the triple-DM equivalent circuit to determine its nine unspecified parameters. Therefore, the key contributions of this paper are:

- An IRMO with QIL strategy is introduced to increase diversity with wider exploration and resilience against local optima.
- The implementation of the proposed IRMO technique extends to three distinct commercial PV systems, of the STM6-40/36, Photowatt PWP201, and R.T.C France cell.
- The IRMO technique demonstrates substantial robustness for both PV models when compared to the RMO and previous results.
- A recognized sustainable improvement for the IRMO is noticed with the increasing number of iterations at each quartile of iterations.
- The average, max, worst values of RMSE are significantly improved when using the IRMO.
- Evaluations of the IRMO algorithm’s effectiveness on the PV triple-DM show a strong correlation between simulated and actual data.

### Paper organization

The paper’s structure includes a problem definition in “PV module parameter extraction model” for the triple-DM frameworks, a detailed description of the IRMO in “Proposed IRMO for PV parameter identification”, thorough examination of experimental findings in “Simulation results and discussions”, and a conclusion in “Conclusion”.

## PV module parameter extraction model

The PV systems are widely regarded as the most prevalent globally due to their to their efficiency in harnessing solar energy for various applications^44,45^. The extraction of parameters from PV models poses a challenging problem, given the characteristics of multi-modality, and nonlinearity. To underscore the I-V characteristics of PV modules, distinct yet comparable circuits have been devised. The triple-DM representation, detailed in^46^, stands out as a highly comprehensive approach, relying on the extraction of nine parameters. In recent decades, the Shockley-diode equivalent circuits have gained popularity as a widely used approximation.

### PV modeling using triple-DM equivalent circuit

**Fig. 1:**
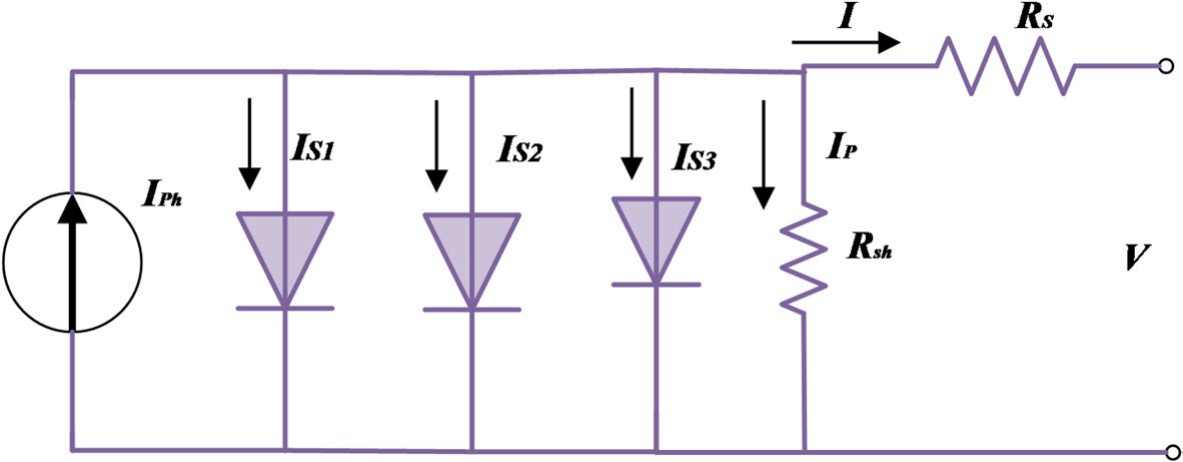
Triple-DM equivalent circuit.

**Fig. 2:**
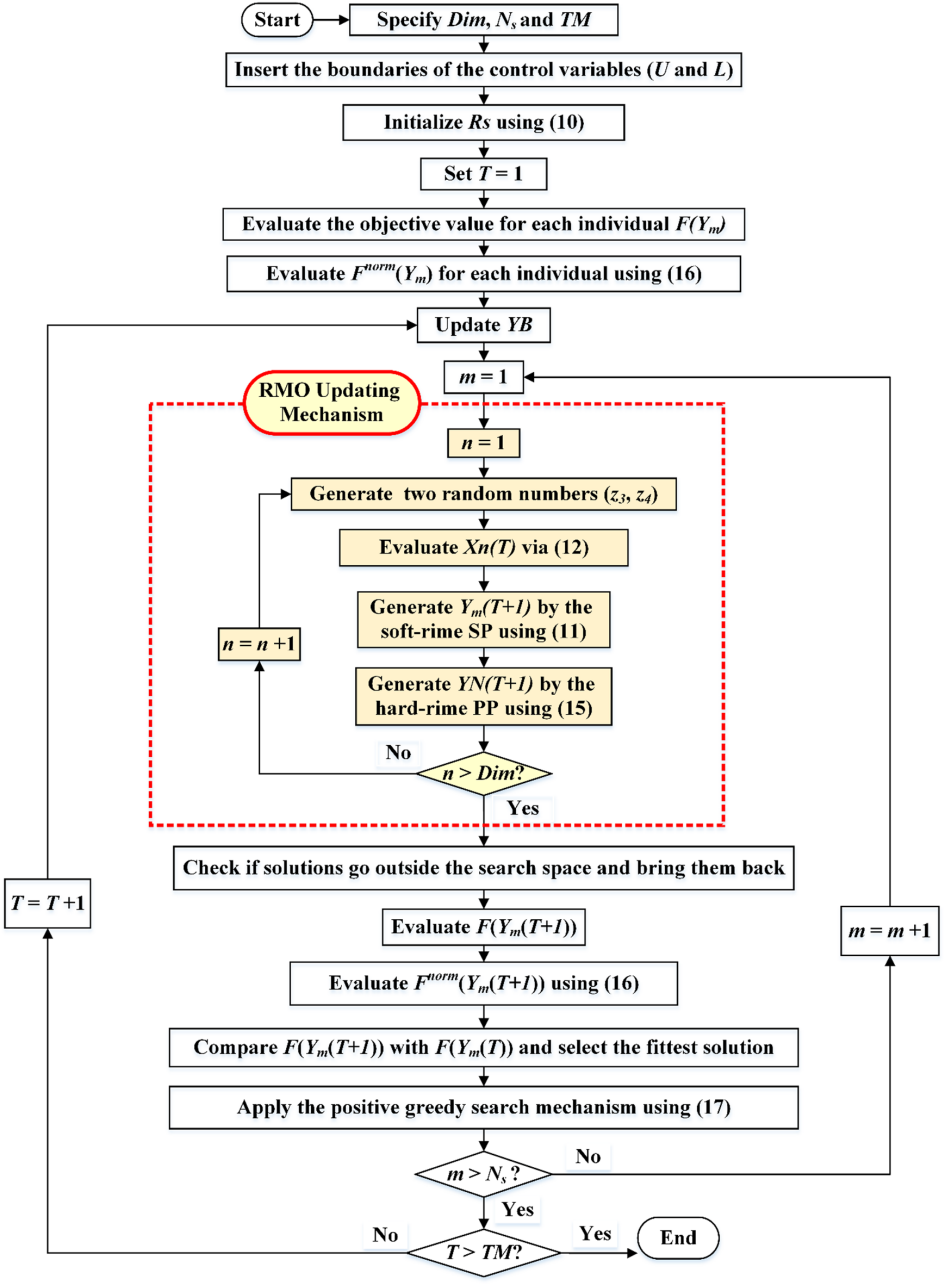
Steps of the standard RMO.

The modeling of PV cells has become an essential tool for exploring the intricate dynamics among various components within a PV system. The triple-DM architecture serves as a widely employed framework for illustrating the characteristics of solar cells. As illustrated in Fig. 1, the triple-DM design presents an equivalent circuit. The core elements encapsulated in PV cells within the triple-DM design comprise three diodes, a current source, and two resistors, as detailed in Fig. 2. The mathematical expression for the load current in the triple-DM design is formally presented in Eq. (1)^47^:

1 $$\:I={I}_{Ph}-{I}_{S1}-{I}_{S2}-{I}_{S3}-{I}_{P}$$

2 $$\:{I}_{S1}={I}_{o1}\times\:\left({e}^{\frac{V+I{R}_{S}}{{\eta\:}_{1}{V}_{{th}}}}-1\right)$$

3 $$\:{I}_{S2}={I}_{o2}\times\:\left({e}^{\frac{V+I{R}_{S}}{{\eta\:}_{2}{V}_{{th}}}}-1\right)$$

4 $$\:{I}_{S3}={I}_{o3}\times\:\left({e}^{\frac{V+I{R}_{S}}{{\eta\:}_{3}{V}_{{th}}}}-1\right)$$

5 $$\:{I}_{P}=\frac{V+I{R}_{S}}{{R}_{sh}}$$

where *I* represents the module’s output current; *I*_*Ph*_ signifies the photocurrent; ‘*I*_*o*1_,’ ‘*I*_*o*2_,’ and ‘*I*_*o*3_’ denote the reverse saturation currents associated, respectively, with the three diodes; ‘*V*’ symbolizes the terminal voltage; ‘*V*_*th*_’ defines the modules’ thermal voltage, as outlined in Eqs. (2–4); ‘*R*_*S*_’ and ‘*R*_*sh*_’ depict the series and shunt resistances that collectively indicate losses in the module; ‘*η*_*1*_,’ ‘*η*_*2*_,’ and ‘*η*_*3*_’ represent the ideality factors pertaining to the three diodes D1, D2, and D3, respectively.

6 $$\:{V}_{th}={K}_{Bolt}\times\:\frac{T}{{q}_{c}}.$$

Furthermore, ‘*K*_*Bolt*_’ characterizes Boltzmann’s coefficient, while ‘*q*_*c*_’ and ‘*T*’ stand for the electron charge and absolute temperature, accordingly^48^.

### PV module model

The expression for the triple-DM equation can be formulated for a PV module comprising *N*_*sh*_ cells in parallel and *N*_*s*_ cells in series. Consequently, Eq. (1) undergoes modification and adaptation:

7 $$\:I={N}_{p}\times\:\left({I}_{Ph}-{I}_{S1}-{I}_{S2}-{I}_{S3}\right)-\frac{V+I\frac{{R}_{S}\times\:{N}_{s}}{{N}_{sh}}}{{N}_{s}{N}_{sh}{R}_{sh}}.$$

Within this context, there is a necessity to estimate nine unknown parameters from the I-V data of PV modules, namely *I*_*Ph*_, *R*_*S*_, *R*_*sh*_, *I*_*S*1_, *η*_1_, *I*_*S*2_, *η*_2_, *I*_*S*3_, and *η*_3_^49,50^.

### Objective function

In the statistical assessment conducted in this investigation, the given formula was employed based on the root mean square error (RMSE)^51^:

8 $$\:RMSE=\sqrt{\frac{1}{{N}_{x}}{\sum\:}_{rd=1}^{{N}_{x}}{\left({I}_{Es,rd}({V}_{Ex,rd},x)-{I}_{Ex,rd}\right)}^{2}}.$$

Here, *N*_*x*_ represents the count of recorded readings, while *I*_*Ex, rd*_ and *V*_*Ex, rd*_ represent the actual measurements of current and voltage for each respective record ‘*rd*’. The solution vector, denoted by x, can be succinctly described as follows:

9 $$\:x=\left[{I}_{ph}\text{}{R}_{sh}{R}_{S}\text{}{I}_{S1}\text{}{\eta\:}_{1}\text{}{I}_{S2}\text{}{\eta\:}_{2}\text{}{I}_{S3}\text{}{\eta\:}_{3}\right].$$

## Proposed IRMO for PV parameter identification

The RMO methodology is grounded in natural occurrences, specifically replicating the growth of rime particles in varying environmental circumstances. This approach imitates the pertinent environmental conditions via the soft-rime and the hard-rime PP^39^. Whereas the hard-rime PP simulates the damaging seen in the formation of rime, where the fitness function is influenced by the efficacy of particle motion, the soft-rime SP simulates the formation of rime components under a breezy climatic situation^52,53^. Additionally, a crossover mechanism is activated to aid in the exchange of information between the particles, fostering enhanced convergence.

### Standard RMO

The rime population is initialised including an arbitrary search procedure, following a conventional method seen in numerous computer algorithms based on populations^54^. During this beginning, the rime position (*Y*_*mn*_) is expressed as follows:

10 $$\:{Y}_{mn}\left(0\right)={L}_{m}+{z}_{1}\left({U}_{m}-{L}_{m}\right);m=1:{N}_{s},n=1:Dim$$

where the population size is indicated by *N*_*s*_; The problem dimensional space is represented by *Dim*; the smallest and greatest dimensional constraints are referred to as *L*_*m*_ and *U*_*m*_; *z*_*1*_ denotes a value from the set (0,1) that was selected at random.

The soft-rime SP updates the position of each rime particle based on two key factors of exploration and randomization. Exploration is implemented where the influence of the best global solution helps guide the particle toward better regions in the search space. Randomization is implemented where an additional perturbation, controlled by the environmental parameters, ensures diverse movement. Therefore, the position of each rime particle, in the subsequent iteration (*T* + 1), can be upgraded via the soft-rime SP as symbolized by *YM*(*T* + 1) as follows:

11 $$\:Y{M}_{mn}(T+1)=Y{B}_{n}\left(T\right)+{X}_{n}\left(T\right),\:if\:{z}_{3}<\sqrt{\frac{T}{TM}}$$

where, *YB*_*n*_*(T)* indicates the dimension (*n*) regarding the best-so-far solution (*YB*) inside the present iteration (*T*) while *TM* refers to its maximum value; *z*_*2*_ denotes randomized selected value in [0,1]; *X*_*n*_*(T)* is an artificial vector that can be generated^55^ as follows:

12 $$\:{X}_{n}\left(T\right)={z}_{3}.\left[H\times\:\left({U}_{n}-{L}_{n}\right)+{L}_{n}\right]\times\:\beta\:\left(T\right){cos}(\theta\:\left(T\right))$$

while *z*_*3*_ denotes randomized selected value in [-1,1], *H* symbolizes the degree of adhesion which is considered as randomized selected value in [0,1]; *β(T)* and *θ(T)* simulates the external circumstances representing the environmental factors which are modelled as follows:

13 $$\:\theta\:\left(T\right)=\left(\frac{0.1\times\:\pi\:\times\:T}{TM}\right)$$

14 $$\:\beta\:\left(T\right)=1-\left({W}^{-1}\times\:\left(round\left(W\times\:T/TM\right)\right)\right).$$

Consequently, as per^39^, the factor “*W*” governs the step function’s division and by defaults to five.

As shown in the soft-rime SP, *β*(*T*) adjusts the step size towards the best-known solution, and *θ*(*T*) adds a stochastic component, preventing premature convergence while *X*_*n*_*(T)* is a randomly generated perturbation factor to introduce exploration. This mechanism allows the rime particles to gradually converge toward optimal solutions while maintaining diversity.

The hard-rime PP reinforces local exploitation by intensifying movement toward the best solution. Unlike SP, which includes randomness for broader search, PP prioritizes refinement by adjusting a particle’s position based on its current fitness level. Therefore, in conditions of high wind, the hard-rime PP is repeated. Therefore, the position of each rime particle can be upgraded via the hard-rime SP as symbolized by *YN*(*T* + 1) as follows^55^:

15 $$\:Y{N}_{mn}\left(T+1\right)=\:\left\{\begin{array}{c}Y{B}_{n}\left(T\right)\:\:\:\:if\:{z}_{4}<{F}^{norm}\left({Y}_{m}\left(T\right)\right)\\\:Y{M}_{mn}\left(T+1\right)\:\:\:\:\:\:Else\:\:\:\:\:\:\:\:\:\:\:\:\:\:\:\:\:\:\:\:\end{array}\right.m=1:{N}_{s},n=1:Dim$$

where *z*_*4*_ denotes randomized selected value in [0, 1]; *F*(*Y*_*m*_(*T*)) indicates the value of fitness functions of the solution particle (*Y*_*m*_(*T*)) while *F*^*norm*^(*Y*_*m*_(*T*)) indicates its associated normalized one as follows:

16 $$\:{F}^{norm}\left({Y}_{m}\right(T\left)\right)=\frac{F\left({Y}_{m}\right(T\left)\right)}{\sqrt{{\sum\:}_{m=1}^{{N}_{s}}{\left(F\left({Y}_{m}\right(T\left)\right)\right)}^{2}}};m=1:{N}_{s}.$$

The process involves assessing fitness values before and after updates using the positive greedy search mechanism. New positions for rime particles are created using either hard-rime PP, soft-rime SP or QIL method. If the updated fitness value is superior, the optimal solution replaces the suboptimal one, enhancing the overall global solution quality. The positive greedy search mechanism in the IRMO ensures that only improved solutions are retained, thereby guiding the optimization process toward better convergence. This mechanism evaluates the newly generated solutions ($\:Y{N}_{m}\left(T+1\right)$) and replaces the current solutions ($\:{Y}_{m}\left(T\right)$) only if they exhibit a better fitness value. Therefore, the positive greedy selection is performed as follows:

17 $$\:Y{N}_{m}\left(T+1\right)=\:\left\{\begin{array}{c}{Y}_{m}\left(T\right)\:\:\:\:\:\:\:\:\:\:\:\:\:\:\:\:\:\:\:\:if\:F\left({Y}_{m}\left(T\right)\right)<F\left({YN}_{m}\left(T+1\right)\right)\\\:Y{N}_{m}\left(T+1\right)\:\:\:\:\:\:\:\:Else\:\:\:\:\:\:\:\:\:\:\:\:\:\:\:\:\:\:\:\:\:\:\:\:\:\:\:\:\:\:\:\:\:\:\:\:\:\:\:\:\:\:\:\:\:\:\:\:\:\:\:\:\:\:\end{array}\right.\:\:\:\:\:\:\:,m=1:{N}_{s}.$$

This technique ensures the population evolves optimally by actively replacing agents during updates. The procedure entails coming up with a fresh particle solution, evaluating its fitness level, and contrasting it with the prior one. If the newly acquired fitness is superior, it will take the place of the subpar solution, improving the quality of the solutions as a whole. Throughout alterations, this process continuously substitutes particles to guarantee beneficial evolution of the population. Figure 2 shows the important phases of the RMO, which are carried out iteratively until a particular amount of iterations (*TM*) is obtained.

### Proposed improved RMO (IRMO) incorporating quadratic interpolation learning (QIL) strategy

In order to improve population variety and strengthen the RMO algorithm’s exploring abilities, this study proposes an IRMO merging with the Quadratic Interpolation Learning (QIL) technique. QIL is a local search approach that fits the structure of a curve to a parabola function with the goal of locating the curve’s extremes. The QIL strategy improves solution refinement by selecting three different rime particles instead of relying solely on the best-known one. This technique provides a more diverse update, reducing stagnation.

The QIL strategy was integrated into the RMO to support the equilibrium between exploitation and exploration, thereby improving solution diversity and search precision. Unlike traditional gradient-based methods that necessitate derivative information, QIL operates without derivatives, making it particularly suitable for complex, non-smooth, or high-dimensional optimization problems^56^. By employing a second-degree polynomial interpolation function, QIL dynamically approximates optimal step sizes, avoiding the limitations of fixed learning rates or arbitrary perturbations. Conventional local search methods, such as hill climbing or greedy algorithms, often focus on incremental improvements, which can lead to premature convergence in multimodal landscapes^57^. QIL addresses this issue by selecting three distinct rime particles for interpolation, rather than relying solely on the current best solution. This approach ensures a more comprehensive sampling of the search space, thereby reducing the risk of stagnation in local optima^56^. Alternative techniques like Gaussian mutation, commonly used in metaheuristics, introduce random perturbations that can result in inefficient exploration due to their stochastic nature^57,58^. In contrast, QIL offers structured and adaptive movements based on interpolation, leading to a more directed and efficient search process. Similarly, strategies such as Lévy flight-based searches, often employed in algorithms like Cuckoo Search^59^ or Whale Optimization Algorithm^60^, may produce excessively large step sizes, causing instability during the fine-tuning phase of optimization. QIL mitigates this by smoothly adjusting step sizes through quadratic interpolation, facilitating controlled and stable convergence. In summary, the integration of QIL into the RMO algorithm provides a derivative-free, adaptive mechanism that enhances both exploration and exploitation capabilities. By dynamically adjusting step sizes and utilizing multiple reference points, QIL offers a more balanced and efficient search strategy compared to traditional local search methods, random perturbations, or Lévy flight-based approaches. The successful integration of QIL into the MGA resulted in notable enhancements in both performance and stability across benchmark models. This integration not only improved the algorithm’s optimization efficiency but also contributed to a substantial reduction in energy losses while promoting environmental sustainability through decreased emissions. The findings highlight the overall practical effectiveness of QIL-enhanced MGA in real-world energy optimization applications, reinforcing its potential for advancing renewable energy solutions and optimizing photovoltaic systems^61^.

It is incorporated into the RMO to add higher diversity and allow non-linear modifications by means of its quadratic operations where it refines solution particle (*Y*_*m*_(*T*)) around itself and two random neighbors (*Y*_*R1*_(*T*) and *Y*_*R2*_(*T*)). Therefore, the position of each rime particle can be upgraded via the QIL strategy as symbolized by *YN*(*T* + 1) as follows:

18 $$\:Y{N}_{m}(T+1)=\frac{A+B+C}{2\times\:\left(a+b+c\right)};\text{}m=1:{N}_{s}$$

where

19 $$\:A=\left({Y}_{R1}{\left(T\right)}^{2}-{Y}_{R2}{\left(T\right)}^{2}\right)\times\:F\left({Y}_{m}\left(T\right)\right)$$

20 $$\:B=\left({Y}_{R2}{\left(T\right)}^{2}-{Y}_{m}{\left(T\right)}^{2}\right)\times\:F\left({Y}_{R1}\left(T\right)\right)$$

21 $$\:C=\left({Y}_{m}{\left(T\right)}^{2}-{Y}_{R1}{\left(T\right)}^{2}\right)\times\:F\left({Y}_{R2}\left(T\right)\right)$$

22 $$\:a=\left({Y}_{R1}\left(T\right)-{Y}_{R2}\left(T\right)\right)\times\:F\left({Y}_{m}\left(T\right)\right)$$

23 $$\:b=\left({Y}_{R2}\left(T\right)-{Y}_{m}\left(T\right)\right)\times\:F\left({Y}_{R1}\left(T\right)\right)$$

24 $$\:c=\left({Y}_{m}\left(T\right)-{Y}_{R1}\left(T\right)\right)\times\:F\left({Y}_{R2}\left(T\right)\right)$$

where *F* (*Y*_*R1*_(*T*)), *F(Y*_*R2*_(*T*)) and *F(Y*_*m*_(*T*)) are the fitness values of, respectively, the current particle and the two other neighbors that are randomly selected.

**Fig. 3:**
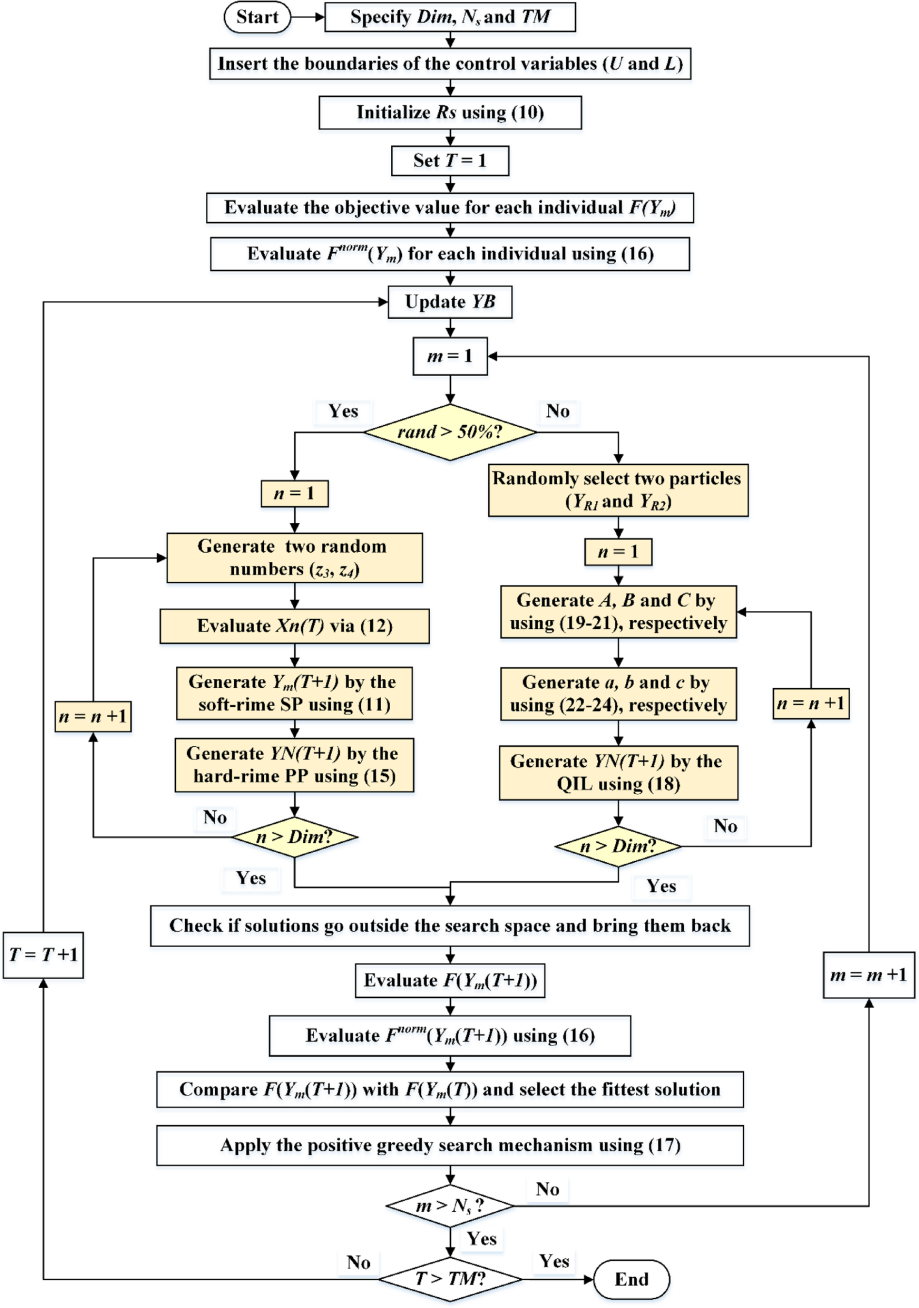
Steps of the IRMO.

The proposed IRMO technique, illustrated in Fig. 3, iteratively executes the entire process until reaching a specified number of iterations (*TM*). Also, a MATLAB code for the IRMO with QIL for addressing the sphere function is displayed in the Appendix. Utilizing the QIL technique or the soft-rime SP and hard-rime PP phases, it updates rime agent positions, calculates fitness values, and performs positive greedy search. As shown in this figure, the proposed IRMO has several main steps as follows:

- **Step 1: Initialization**: In this step, the search space is defined, and the initial population of rime particles is arbitrarily created inside the problem’s dimensional constraints. Also, the algorithm parameters are set, including maximum iterations and population size.
- **Step 2: Fitness Evaluation**: The RMSE is utilized to evaluate the quality of each rime particle’s position in the search space.
- **Step 3: Soft-Rime SP**: The rime particles’ positions are updated based on environmental factors.
- **Step 4: Hard-Rime PP**: The local search of the rime particles’ positions is enhanced by refining solutions through structured perturbation.
- **Step 5: QIL Strategy**: Three distinct rime particles are selected instead of only the best one, allowing a more diversified exploration approach. Then, quadratic interpolation is applied to refine particle movement and balance global exploration with local exploitation.
- **Step 6: Greedy Selection Mechanism**: The RMSE of new and old solutions are contrasted. The solution with the best fitness for the next iteration is retained, ensuring continual population improvement.
- **Step 7: Convergence Check**: The steps 3–6 are repeated iteratively until the stopping criterion is reached.
- **Step 8: Optimal Solution Extraction**: The best rime particle (solution) is extracted corresponding to the optimal PV model parameters.

The computational complexity of the IRMO depends on the key operations performed during each iteration. The main computational steps contributing to the overall complexity depend on the population initialization, fitness evaluation, Soft-rime SP and hard-rime PP, QIL and greedy selection. The algorithm initializes a population of *N*_*s*_ rime particles, each with d dimensions (search variables). Thus, the initialization step has a complexity of O(*N*_*s*_×*Dim*). The algorithm evaluates the fitness function (RMSE) for all *N*_*s*_ particles. The complexity of the fitness function evaluation depends on its mathematical formulation, denoted as O(*F*(*Dim*)). Every particle upgrades its position depending on either the soft-rime SP, hard-rime PP or QIL in a separate way. Since each of the *N*_*s*_ particles updates d dimensions, this step runs in O(*N*_*s*_×*Dim*). As the greedy selection mechanism compares new and old fitness values and updates the best solution. Since this requires N comparisons, it runs in O(*N*_*s*_). Since all the above steps are repeated for *TM* iterations, the total complexity of IRMO is O(*N*_*s*_× *TM*×*Dim*). On the other side, the original RMO algorithm has a similar updating mechanism but lacks QIL which is either activated or not instead of soft-rime SP or hard-rime PP. Since the QIL step operates in O(*N*_*s*_×*Dim*), it does not significantly increase the overall complexity. Thus, it has a similar complexity of O(*N*_*s*_× *TM*×*Dim*). However, IRMO improves convergence speed, potentially reducing the required number of iterations, making it computationally more efficient in practice.

## Simulation results and discussions

In this paper, the proposed IRMO method is expanded to estimate PV parameters for three different PV systems: STM6-40/36 module, Photowatt PWP201 module, and R.T.C France cell. The STM6-40/36 system consists of 36 series-connected monocrystalline cells, each measuring 38 mm x 128 mm, operating under 1000 W/m^2^ irradiation at a temperature of 51 °C^62^. The PWP201 system features 36 series-connected silicon cells of polycrystalline type at an irradiance of 1000 W/m^2^ and a temperature of 45 °C^63^. Finally, the R.T.C France cell operates at 1000 W/m^2^ sun irradiance and 33 degrees Celsius temperature.

### Simulation results regarding STM6-40/36 PV module

**Fig. 4:**
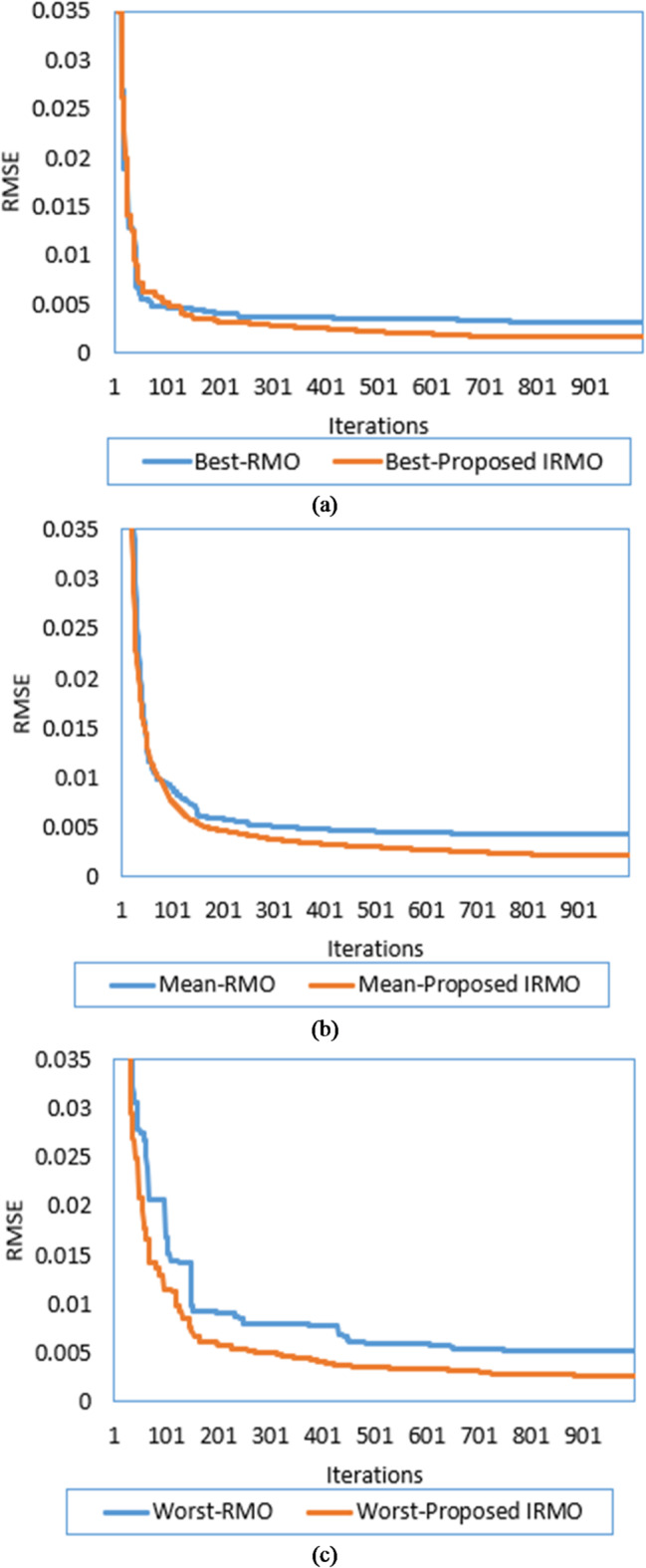
Best, mean, and worst convergence properties of RMO and IRMO for STM6-40/36 PV Module. (a) Best convergence properties. (b) Mean convergence properties. (c) Worst convergence properties.

By implementing both the original RMO and the novel IRMO, the properties of the triple-DM of the STM6-40/36 PV module are determined. The related variables and findings are outlined in Table 1. As illustrated from this table, the original RMO yields an RMSE value of 0.00354, whereas the novel IRMO achieves the minimum RMSE value of 0.001689. Thirty separate runs are used to evaluate the efficacy of both the novel IRMO and the original RMO. Their convergence properties at their best, medium, and worst can be observed in Fig. 4.

**Fig. 5:**
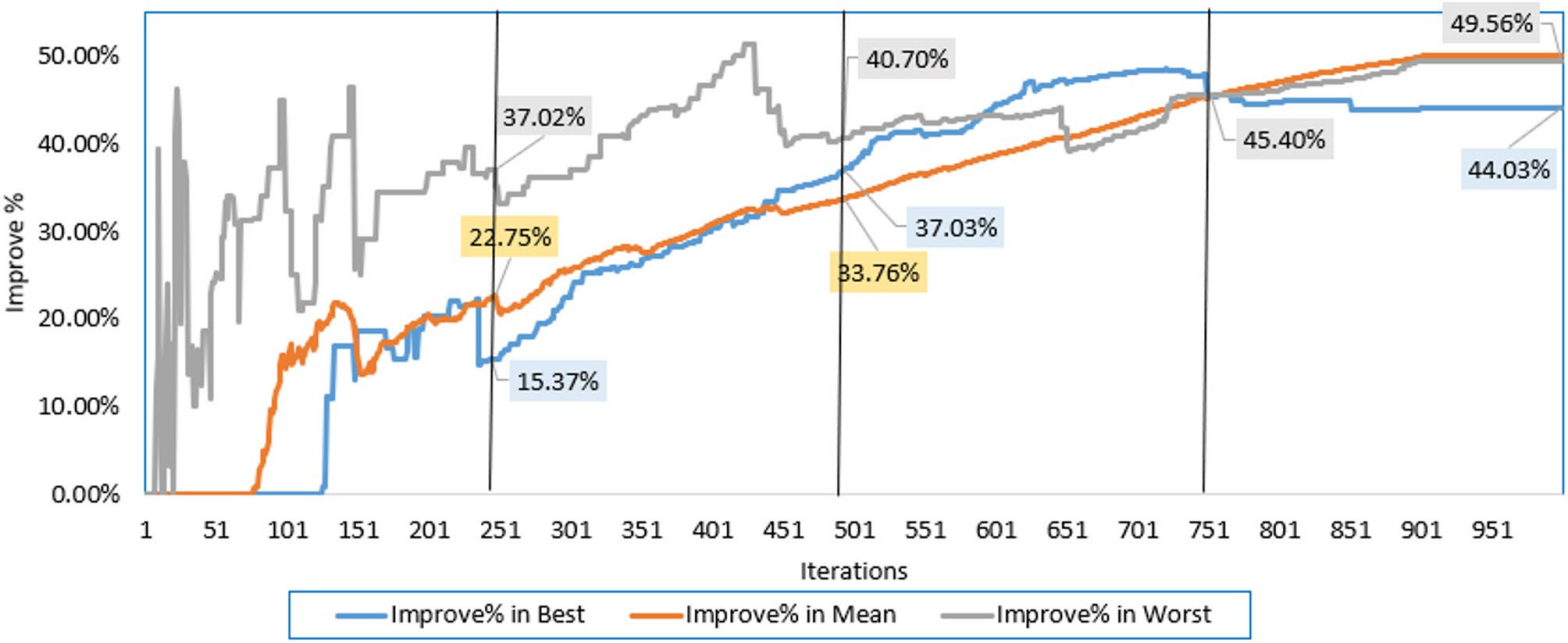
Improvement percentage of IRMO compared to RMO in their best, mean, and worst convergences.

Additionally, the electrical elements correlated with the proposed IRMO tend to be as follows: 16.91705 Ω and 0.004643 Ω for the shunt and series resistances; 1.944, 1.973, and 1.464 for the ideality factors of D1, D2, and D3; 1.66348223 A for the photocurrent; 4.59415E-01 µA, 3.5926 µA, and 9.294E-01 µA for the reverse saturation currents for D1, D2, and D3. The novel IRMO shows substantial enhancement from the beginning of the iterations, as shown in Fig. 5. The reason for this is that the LEO mechanism activates an enhanced exploration potential. The enhancement begins at the 60th iteration for the best convergence characteristics, but for the average and worst characteristics, the progress becomes noticeable earlier.

**Table 1:** STM6-40/36 PV parameters employing RMO and IRMO.

Item	Lower bound	Upper bound	RMO	IRMO
*I*_*Ph*_ (A)	0.00	2.00	1.65874249	1.66348223
*R*_*s*_ (Ω)	0.00	0.36	0.00158282	0.004643798
*R*_*sh*_ (Ω)	0.00	1000	30.4468306	16.91705038
*I*_*S1*_ (A)	0.00	50E−06	9.6845E−07	4.59415E−07
*I*_*S2*_ (A)	0.00	50E−06	3.4417E−09	3.5926E−06
*I*_*S3*_ (A)	0.00	50E−06	3.3729E−06	9.29455E−07
*η*_*1*_	1.00	2.00	1.70404127	1.944427681
*η*_*2*_	1.00	2.00	1.79899262	1.973872807
*η*_*3*_	1.00	2.00	1.61291207	1.464785202
RMSE	–	–	0.00354	0.001689

**Fig. 6:**
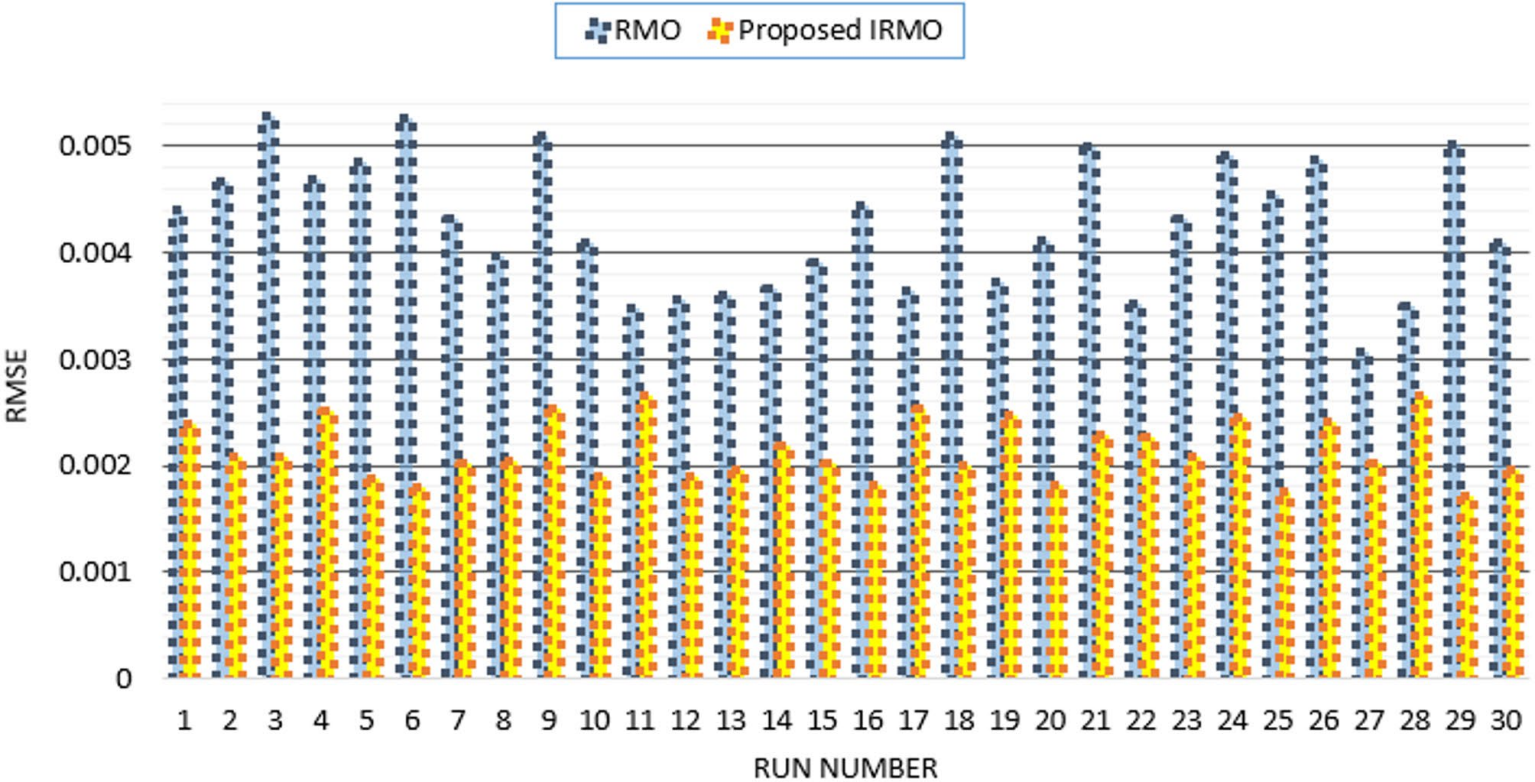
Achieved RMSE by RMO and IRMO in all runs for STM6-40/36 PV module.

**Fig. 7:**
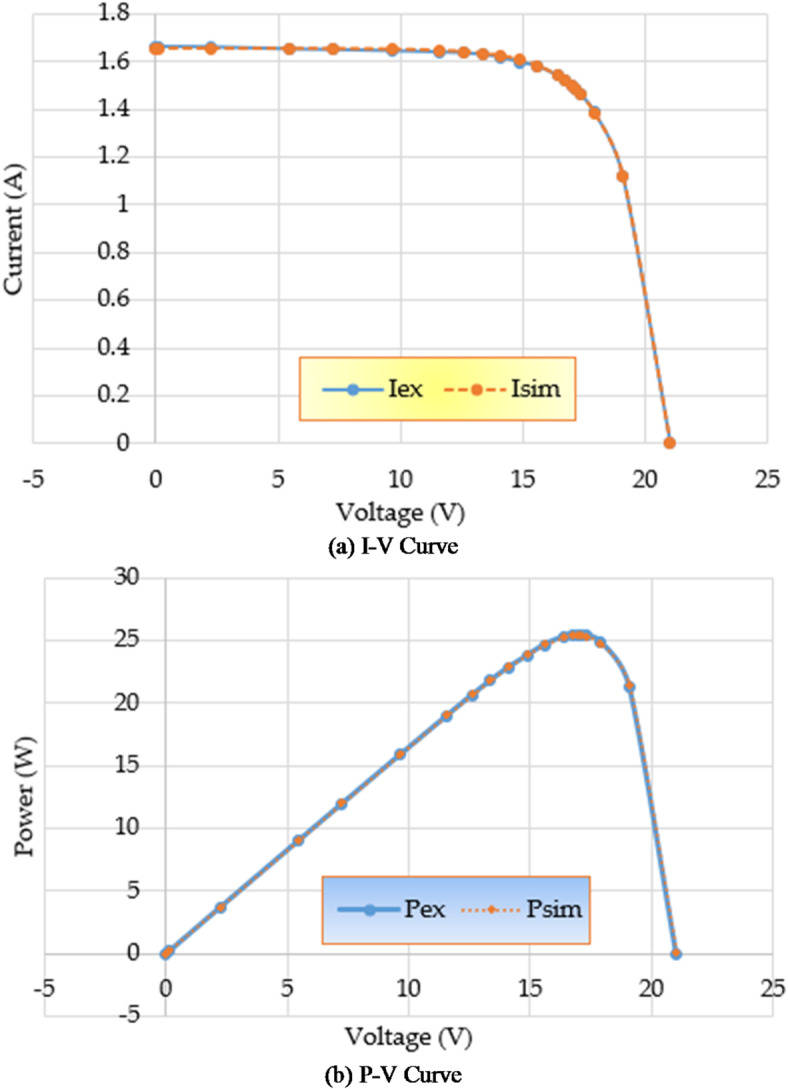
Estimated data by means of the proposed IRMO versus the measured data for STM6-40/36 PV module.

Furthermore, the disparity in RMSE observed for each run that was computed using RMO and IRMO can be seen in Fig. 6, along with the corresponding enhancement of using the novel IRMO instead of RMO. It is demonstrated that the novel IRMO outperforms the RMO in terms of robustness and superiority. It displays a 49.56% average improvement rate, with minimum and maximum improvements of 44.03% and 45.40%, respectively. Furthermore, Fig. 7 contrasts the data used for parameter estimation with the simulated efficiency of the P-V and I-V features using the outcomes of the triple-DM design. The curves provided manifest a significant efficient correlation between the observed and calculated PV parameters using the proposed IRMO.

Furthermore, a comparison between the original RMO, IRMO, as well as other recently developed techniques are clarified in Table 2 for this model. These recently developed techniques are teaching learning studying-based Approach (TLSBA)^65–67^, social network searching (SNS)^64^, the tuna swarm method (TSM)^68^, artificial electric field optimizer (AEFO)^69^, African Vultures Optimization (AVO)^70^ and artificial hummingbird optimizer (AHO)^71^. The RMSE values for the Min, Max, Mean, and Standard Deviation are 1.7192E-03, 2.6632E-03, 2.1429E-03, and 2.8467E-04, respectively, according to the information in this table. The obtained results demonstrate that the employed IRMO boosts significant enhancements in accuracy and effectiveness for best triple-DM characterization when compared to other recently developed competing solutions.

**Table 2:** RMO and IRMO against reported results for STM6-40/36 PV Module.

Optimizer	Min (RMSE)	Mean (RMSE)	Max (RMSE)	Std (RMSE)
AEFO [70]	1.7203E−3	–	–	–
SNS [73]	2.30797E−3	2.829747E−3	3.34643E−3	3.3307E−4
TSLBA [72]	2.2864E−3	3.4767E−3	4.982E−3	6.4695E−4
AVO [72]	3.5398E−3	4.5393E−3	5.976E−3	6.4884E−4
Original RMO	3.0715E−03	4.2909E−03	5.2799E−03	6.3159E−04
Proposed IRMO	1.7192E−03	2.1429E−03	2.6632E−03	2.8467E−04

### Simulation results regarding photowatt PWP201 PV module

This module is utilized to extract the triple-DM characteristics using the novel IRMO and the original RMO. The related variables and findings are outlined in Table 3. As illustrated from this table, the original RMO yields an RMSE value of 0.00315355, whereas the novel IRMO finds minimal RMSE value of 0.002428759. Additionally, the electrical elements correlated with the proposed IRMO tend to be as follows: 27.38819135 Ω and 0.033369135 Ω for the shunt and series resistances; 1.96258, 1.642196, and 1.34957 for the ideality factors of D1, D2, and D3; 1.66348223 A for the photocurrent; 0 µA, 2.14701E-01 µA, and 3.40761 µA for the reverse saturation currents for D1, D2, and D3.

**Fig. 8:**
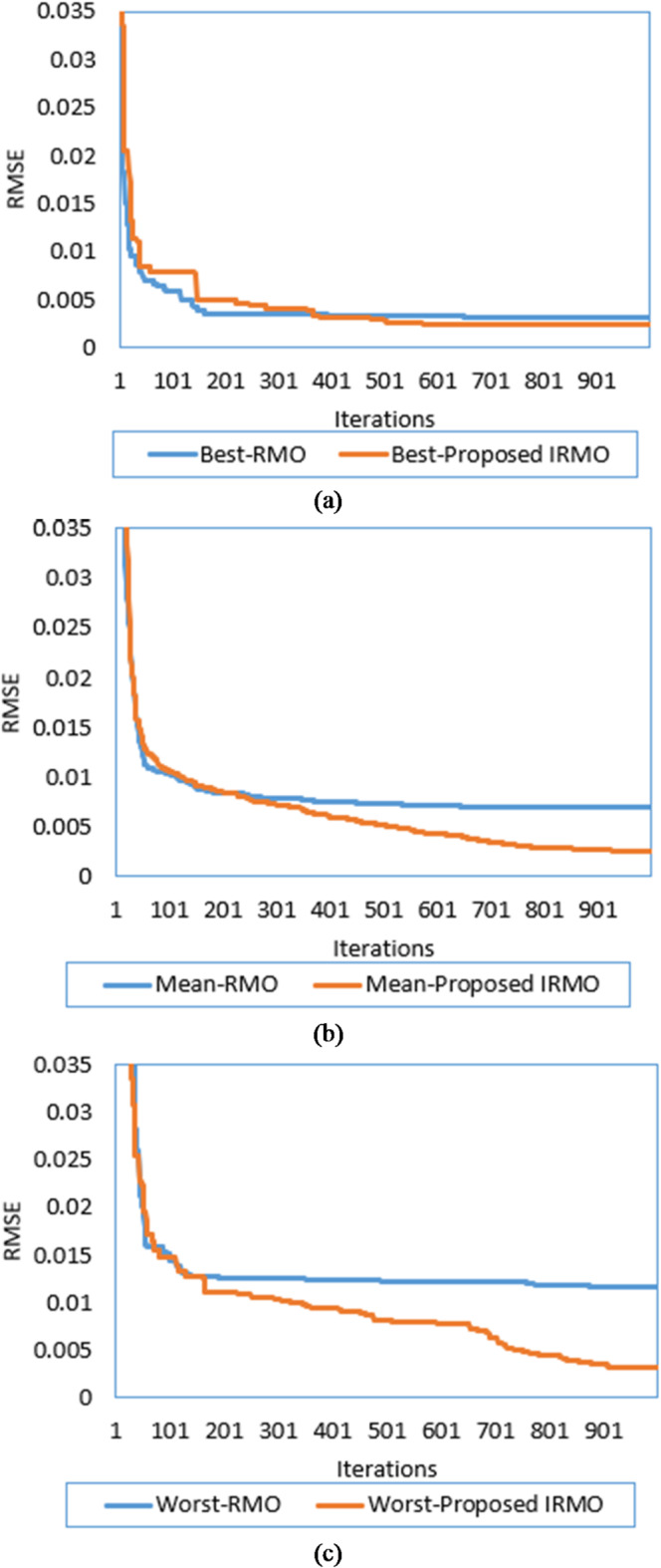
Best, mean, and worst convergence properties of RMO and IRMO for PWP201 PV module. (**a**) Best convergence properties. (**b**) Mean convergence properties. (**c**) Worst convergence properties.

**Fig. 9:**
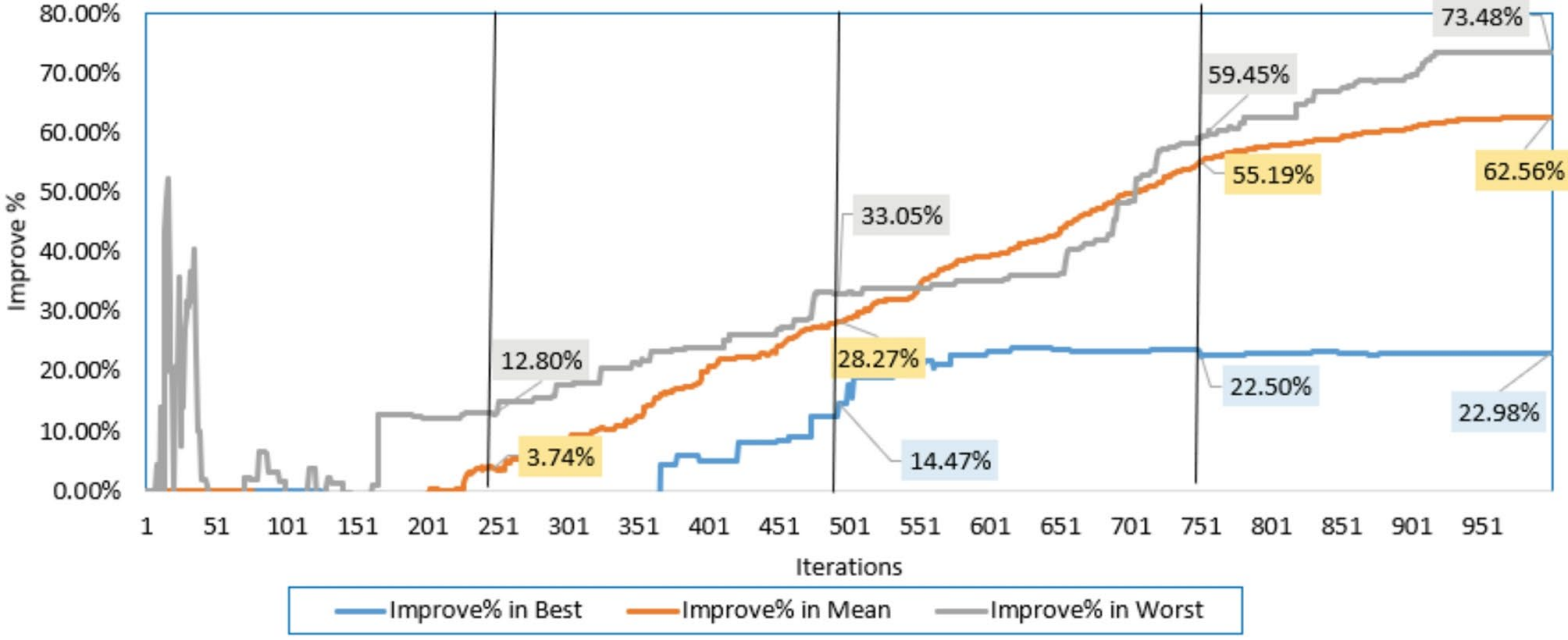
Improvement percentage of IRMO compared to RMO in their best, mean, and worst convergences.

A substantial enhancement can be attributed to the novel IRMO from the start of the iterative journey, as manifested in Figs. 8 and 9. The reason for this is that the LEO mechanism activates an enhanced exploration potential. The enhancement begins after the 120 iterations for the best convergence characteristics, but for the average and worst characteristics, the progress becomes noticeable earlier. It is demonstrated that the novel IRMO outperforms the RMO in terms of robustness and superiority. It shows an average improvement of 62.56%, ranging from 22.98 to 73.48%.

**Table 3:** PWP201 PV parameters employing RMO and IRMO.

Item	Lower bound	Upper bound	RMO	IRMO
*I*_*Ph*_ (A)	0.00	2.00	1.02938243	1.030493996
*R*_*s*_ (Ω)	0.00	2.00	0.03232178	0.033369135
*R*_*sh*_ (Ω)	0.00	2000.00	53.5570524	27.38819135
*I*_*S1*_ (A)	0.00	50E−06	2.4446E−06	0
*I*_*S2*_ (A)	0.00	50E−06	5.6441E−06	2.14701E−07
*I*_*S3*_ (A)	0.00	50E−06	1.9005E−05	3.40761E−06
*η*_*1*_	1.00	2.00	1.32738853	1.962585395
*η*_*2*_	1.00	2.00	1.97838278	1.642196323
*η*_*3*_	1.00	2.00	1.9904139	1.349574121
RMSE	–	–	0.00315355	0.002428759

**Fig. 10:**
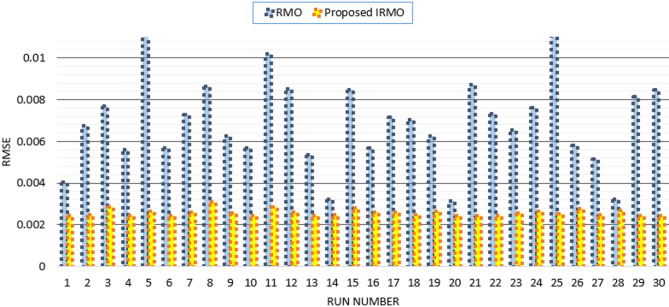
Achieved RMSE by RMO and IRMO in all runs for PWP201 PV Module.

Thirty separate runs are used to evaluate the efficacy of both the novel IRMO and the original RMO. Their convergence properties at their best, medium, and worst can be observed in Fig. 10. The disparity in RMSE observed for each run that was computed using RMO and IRMO can be seen in this figure, along with the corresponding enhancement of using the novel IRMO instead of RMO.

**Fig. 11:**
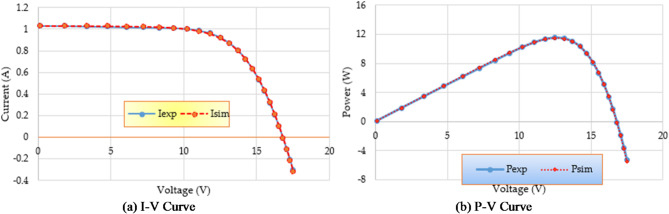
Estimated data by means of the proposed IRMO versus the measured data for PWP201 PV Module.

In addition, Fig. 11 contrasts parameter estimation data with simulated efficiency of P-V and I-V features using triple-DM design outcomes, showing strong correlation between calculated and observed PV parameters using IRMO.

Furthermore, a comparison between the original RMO, proposed IRMO, as well as other recently developed techniques are clarified in Table 4 for this model. These recently developed techniques are Sunflower optimization (SFO)^47^, Cuckoo Search Algorithm (CSA)^73^, PSO^17^, SNS^72^, Biogeography-based Heterogeneous Cuckoo Search (BHCS)^74^, and Artificial ecosystem-based optimizer (AEO)^75^. The RMSE values for the Min, Max, Mean, and Standard Deviation are 3.111E-03, 2.429E-03, 2.584E-03, and 1.631E-04, respectively, according to the information in this table. The employed IRMO boosts significant enhancements in accuracy and effectiveness for the best triple-DM characterization when contrasted to other recently solutions.

**Table 4:** Comparisons of RMO and IRMO versus reported results for PWP201 PV.

Method	Min (RMSE)	Mean (RMSE)	Max (RMSE)	Std (RMSE)
BHCS^74^	3.6790E−03	–	–	–
AEO^75^	2.4800E−03	–	–	–
SFO^47^	8.2500E−02	–	–	–
CSA^73^	3.2000E−03	–	–	–
PSO^17^	3.3925E−3	2.081E−2	3.374E−2	–
SNS^72^	2.5090E−03	3.191E−03	5.511E−03	2.509E−03
RMO	1.173E−02	6.901E−03	3.154E−03	2.159E−03
Proposed IRMO	3.111E−03	2.584E−03	2.429E−03	1.631E−04

### R.T.C France PV cell

**Fig. 12:**
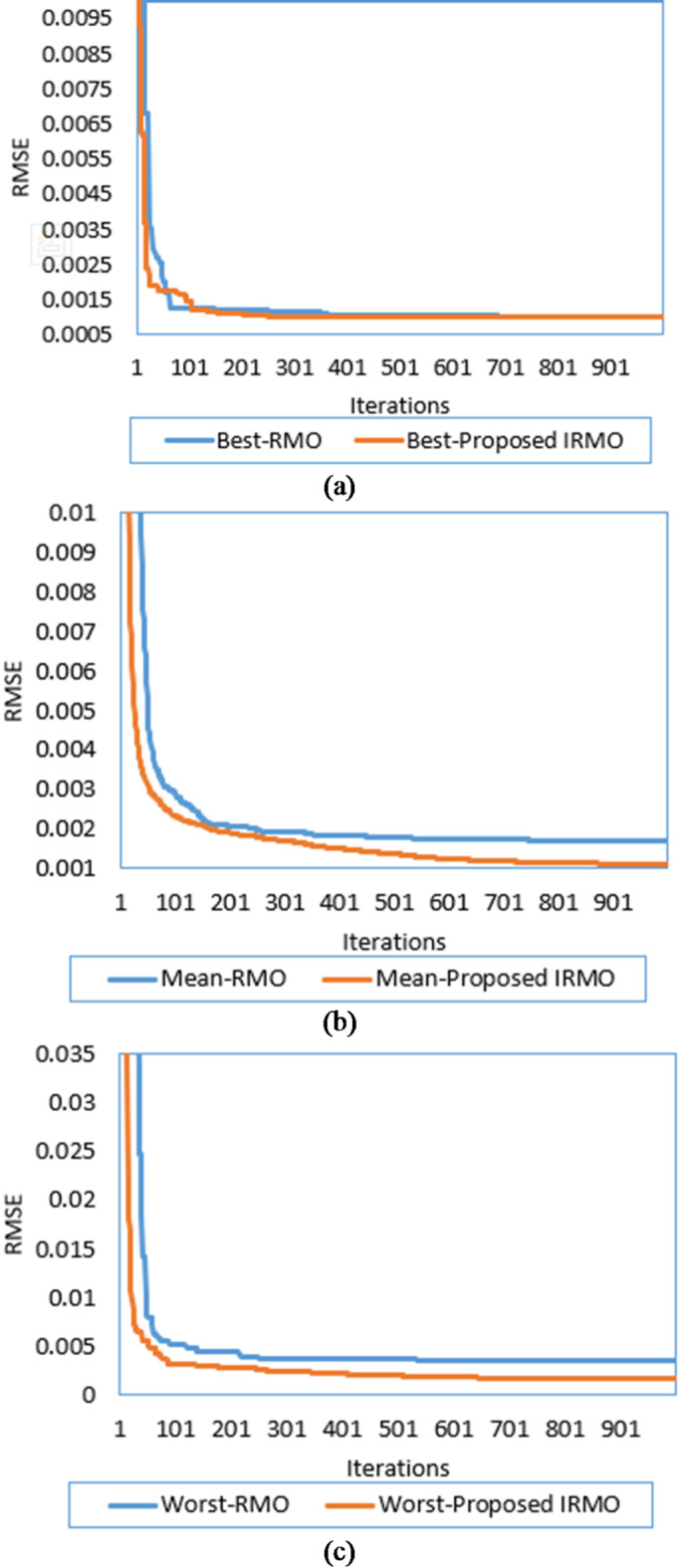
Best, mean, and worst convergence properties of RMO and IRMO for R.T.C France PV Cell considering triple-DM. (**a**) Best convergence properties. (**b**) Mean convergence properties. (**c**) Worst convergence properties.

**Fig. 13:**
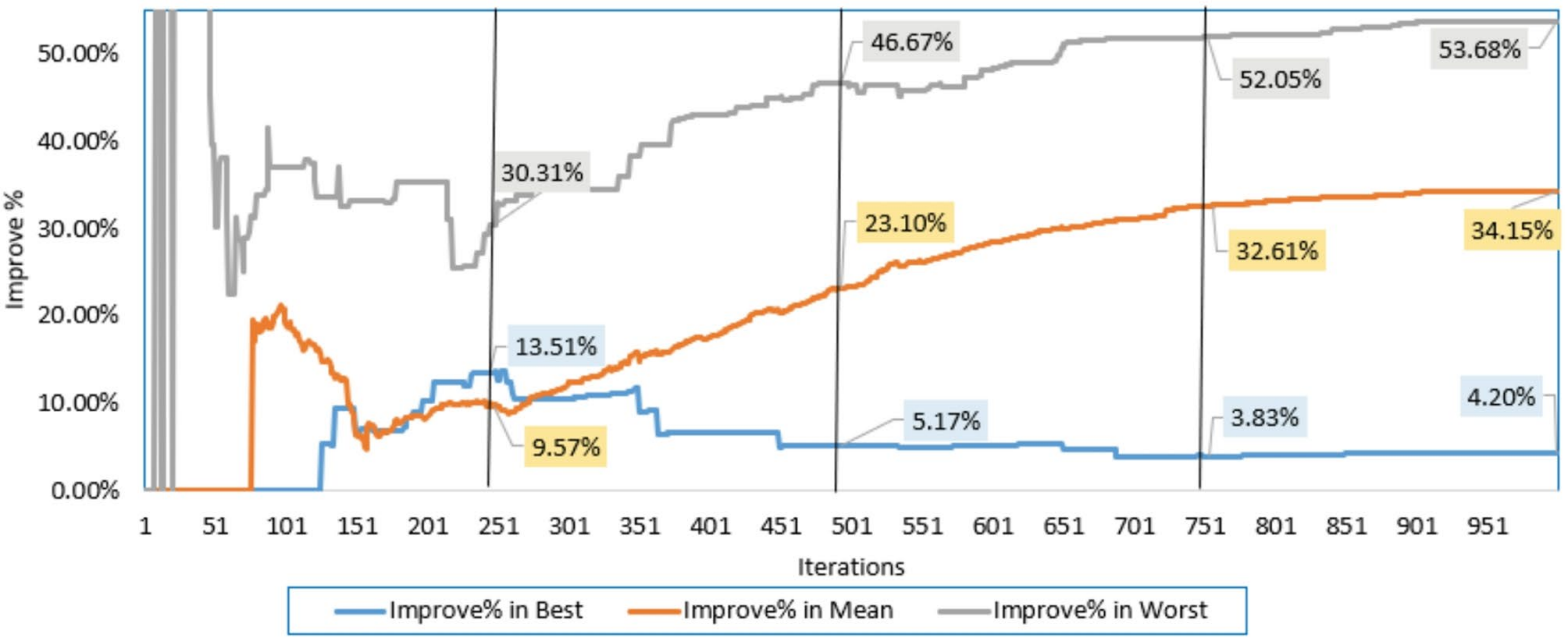
Improvement percentage of IRMO compared RMO in their best, mean, and worst convergences.

It is utilized to extract the one-DM, two-DM, and triple-DM characteristics using the novel IRMO and the original RMO. The related variables and findings for the three models are outlined in Table 5. Moreover, it shows the RMSE of the novel IRMO and the original RMO for the one-DM and the two-DM, where the RMSE values of RMO and the IRMO for the one-DM are 0.00099755 and0.00098613, respectively, and for the two-DM are0.000993817 and 0.000983333, respectively. These results demonstrate the superiority and effectiveness of the novel IRMO compared to the original RMO. As illustrated from this table, the original RMO yields an RMSE value of 0.001030096 for the triple-DM, whereas the novel IRMO specified minimum RMSE value of 0.000987. Additionally, the electrical elements for the triple-DM that are correlated with the proposed IRMO tend to be as follows: 54.44781 Ω and 0.03633 Ω for the shunt and series resistances; 1.904945, 1.482333, and 1.904091 for the ideality factors of D1, D2, and D3; 0.760717 A for the photocurrent; 2.42E-09 µA, 3.27E-07 µA, and 2.76E-09 µA for the reverse saturation currents for D1, D2, and D3. Their convergence properties at their best, medium, and worst can be observed in Fig. 12 while Fig. 13 depicts the improvement percentage of IRMO compared RMO in their best, mean, and worst convergences. In the triple-DM, the novel IRMO displays a 34.15% average improvement rate, with minimum and maximum improvements of 13.52% and 53.68%, respectively. A substantial improvement can be attributed to the novel IRMO from the start of the iterative journey, as manifested in the mentioned figure. The enhancement begins after the 70 iterations for the best convergence characteristics, but for the average and worst characteristics, the progress is noticeable earlier.

**Table 5:** R.T.C France PV parameters employing RMO and IRMO considering one-DM, two-DM and triple-DM.

Item	Lower limit	Upper limit	One-DM		Two-DM		Triple-DM	
			RMO	Proposed IRMO	RMO	Proposed IRMO	RMO	Proposed IRMO
*I*_*Ph*_ (A)	0.00	1.00	0.76055716	0.760784911	0.760864277	0.760746515	0.76096794	0.760717
*R*_*s*_ (Ω)	0.00	0.50	0.03625763	0.036406955	0.036173672	0.036632137	0.037300892	0.03633
*R*_*sh*_ (Ω)	0.00	100.00	57.372545	53.56148396	53.58354831	55.72573648	50.75232738	54.44781
*I*_*S1*_ (A)	0.00	10E−06	3.3687E−07	3.2093E−07	4.3113E−08	6.19476E−07	5.29424E−08	2.42E−09
*I*_*S2*_ (A)	0.00	10E−06	–	–	3.25421E−07	2.4016E−07	1.7659E−07	3.27E−07
*I*_*S3*_ (A)	0.00	10E−06	–	–	–	–	0.000001	2.76E−09
*η*_*1*_	1.00	2.00	1.48537668	1.480528247	1.827202939	1.987334194	1.367966883	1.904945
*η*_*2*_	1.00	2.00	–	–	1.482783518	1.456340137	1.519524163	1.482333
*η*_*3*_	1.00	2.00	–	–	–	–	1.995991274	1.904091
RMSE	–	–	0.00099755	0.00098613	0.000993817	0.000983333	0.001030096	0.000987

**Fig. 14:**
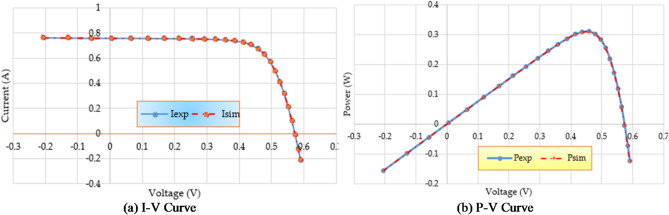
Estimated data by IRMO versus the measured data for R.T.C France PV cell considering triple-DM.

**Fig. 15:**
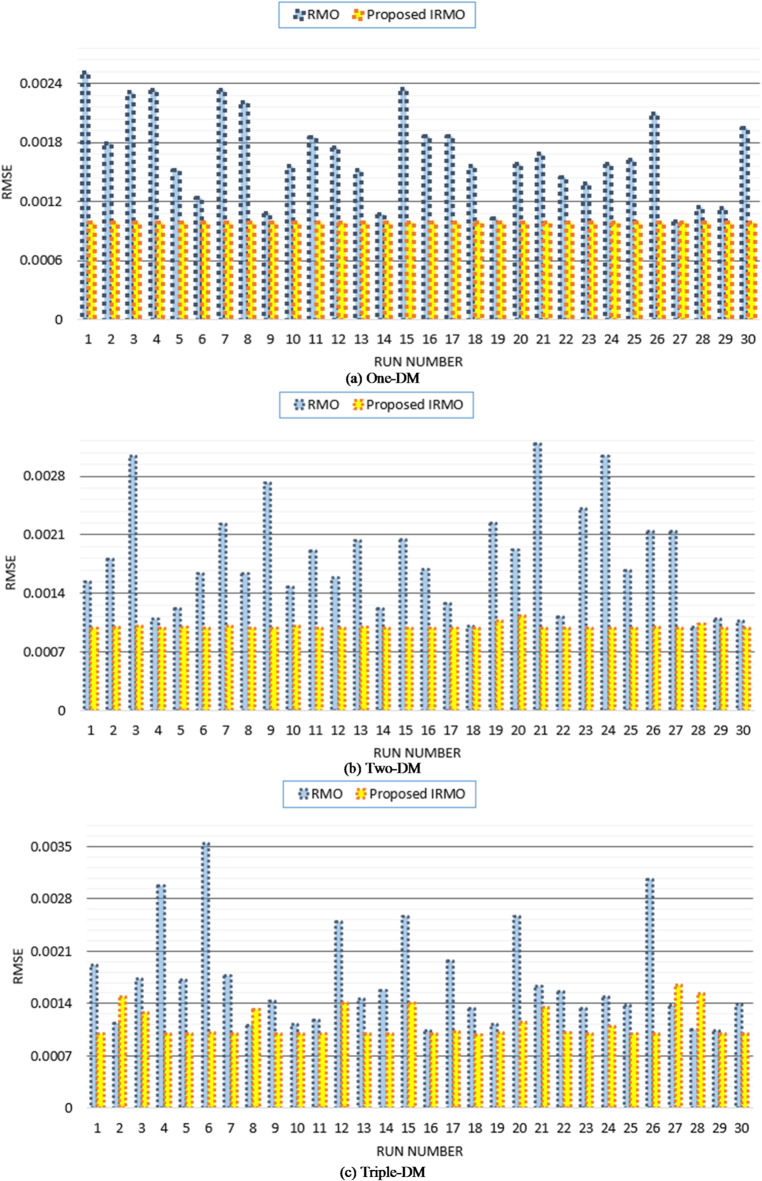
Achieved RMSE by RMO and IRMO in all runs for R.T.C France PV cell considering one-DM, two-DM and triple-DM.

Figure 14 compares the data used for parameter estimation with the simulated P-V and I-V characteristics based on the triple-DM design outcomes. The provided characteristics derive a strong effective correlation between the observed and calculated PV parameters using the proposed IRMO. Furthermore, the disparity in RMSE observed for each run that is computed using RMO and IRMO for the one, two, and triple-DMs of R.T.C France PV cell can be illustrated in Fig. 15, along with the corresponding enhancement of using the novel IRMO instead of RMO.

Furthermore, a comparison between the original RMO, proposed IRMO, as well as other recently developed techniques are clarified in Table 6 for this model. These recently developed techniques are Hazelnut tree search (HTS) algorithm^76^, Artificial bee colony (ABC)^77^, Energy valley optimizer (EVO)^76^, Growth optimizer (GO)^76^, Teaching–Learning–based ABC (TLbABC)^78^, Five Phases Algorithm (FPA)^76^, Flower Pollination Optimizer (FPO)^79^, Sine cosine approach (SCA)^80^, TLBO^81^, Cat Swarm Algorithm (CSA)^82^, Comprehensive learning PSO^83^, and Generalized oppositional TLBO^84^. The RMSE for the Min, Max, Mean, and Standard Deviation are 9.86812 E-04, 1.6436 E-03, 1.12068 E-03, and 2.00229E-04, respectively, according to the information in this table. The employed IRMO boosts significant enhancements in accuracy and effectiveness for best triple-DM characterization.

**Table 6:** Comparisons of RMO and IRMO versus reported results for R.T.C France PV cell considering triple-DM.

Method	RMSE			
	Max	Mean	Min	Std
HTS^76^	1.9880E−03	1.5560E−03	1.1860E−03	2.810E−04
GO^76^	1.0020E−03	9.9793E−04	9.8393E−04	6.4133E−06
FPA^76^	1.4310E−03	1.26510E−03	1.10830E−03	1.1270E−04
EVO^76^	5.3610E−03	2.3850E−03	1.0830E−03	1.6440E−03
TLbABC^78^	–	–	1.50480E−03	–
SCA^80^	–	–	9.86863E−04	–
FPO^79^	–	–	1.93434E−03	–
TLBO^81^	–	–	1.52057E−03	–
CSA^82^	–	–	1.22E−03	–
ABC^77^	–	–	1.2848E−03	–
Comprehensive learning PSO^83^	–	–	1.3991E−03	–
Generalized opposition TLBO^84^	–	–	4.4321E−03	–
RMO	3.54822 E−03	1.70195E−03	1.030096 E−03	6.66011E−04
Proposed IRMO	1.6436 E−03	1.12068 E−03	9.86812 E−04	2.00229E−04

## Conclusion

This study developed a novel enhanced IRMO for the best determination of PV module properties. The novel IRMO is used and evaluated to determine the nine parameters from the PV triple-DM while taking into account three distinct practical PV modules. The novel enhanced IRMO is assessed by combining original RMO with the Quadratic Interpolation Learning (QIL) technique. An enhanced exploration, and resilience against local optima are provided by the incorporation of the QIL approach into the RMO technique. In accordance with IRMO, the exploitation strategy is enabled about approximately 50% of the searching rime particles in each iteration at the start of the iteration process. The novel IRMO is tested using the R.T.C. France, PWP201, and STM6-40/36. In comparison to the original RMO, the novel IRMO significantly improved the precision of identifying the PV parameters with reference to the triple-DM formulation. The superiority of the proposed IRMO method is further asserted through statistical analysis, demonstrating its enhanced accuracy and consistency across different PV modules. For the STM6-40/36 module, the IRMO achieves a mean RMSE of 2.1429E-03 with a standard deviation of 2.8467E-04, while for the Photowatt PWP201 module, it attains a mean RMSE of 2.584E-03 and a standard deviation of 1.631E-04. Similarly, for the R.T.C France PV cell (triple-DM model), the IRMO achieves a mean RMSE of 1.12068E-03 with a standard deviation of 2.00229E-04. These results indicate not only a significant reduction in RMSE but also a lower variability compared to existing techniques, reinforcing IRMO’s robustness and reliability in PV parameter estimation. The proposed IRMO demonstrates greater accuracy and coherence between the calculated and observed values of the P-V and I-V curves for the three PV modules under investigation. Furthermore, it asserts a strong superiority and consistency over previously published results.

### Implications of the IRMO in the field of PV parameter Estimation

The IRMO, by integrating QIL with Rime Optimization, significantly advances PV parameter estimation by enhancing solution diversity, mitigating local optima trapping, and improving convergence accuracy. These improvements have direct implications for PV system modeling, where precise parameter extraction is essential for optimizing fault diagnosis, maximum power point tracking (MPPT), and forecasting. By achieving a lower RMSE across multiple PV modules, IRMO ensures more reliable and adaptable PV models under varying environmental conditions. Additionally, its superior accuracy and robustness make it a valuable tool in real-time PV system monitoring and control, ultimately contributing to improved efficiency and integration of solar energy into modern power grids.

### Future improvements suggestion

While the IRMO method demonstrates superior accuracy and robustness in PV parameter estimation, certain limitations remain. The algorithm’s computational complexity may increase with a higher number of parameters, potentially leading to longer processing times for large-scale PV systems. Additionally, while IRMO effectively mitigates local optima trapping, its performance under extreme environmental variations, such as rapid temperature fluctuations or partial shading, requires further investigation. Future improvements could focus on hybridizing IRMO with adaptive learning strategies to enhance its real-time applicability, integrating physics-informed constraints to improve generalization, and optimizing its computational efficiency for large-scale solar farms. Furthermore, experimental validation with real-time PV data would strengthen its practical relevance and implementation in smart grid applications.

## Electronic supplementary material

Below is the link to the electronic supplementary material.

Supplementary Material 1

## Data Availability

Data is contained within the article.

## References

[1] Abdelaal, A. K. & El-Fergany, A. Estimation of optimal Tilt angles for photovoltaic panels in Egypt with experimental verifications. *Sci. Rep.* **13** (1). 10.1038/s41598-023-30375-8 (2023). 10.1038/s41598-023-30375-8 PMC9968325 36841921

[2] Abdelaal, A. K., Alhamahmy, A. I. A., Attia, H. E. D. & El-Fergany, A. A. Maximizing solar radiations of PV panels using artificial gorilla troops reinforced by experimental investigations. *Sci. Rep.* **14** (1). 10.1038/s41598-024-53873-9 (2024). 10.1038/s41598-024-53873-9 PMC10861506 38347025

[3] Sharma, A. et al. Performance investigation of state-of-the-art metaheuristic techniques for parameter extraction of solar cells/module. *Sci. Rep.* **13** (1). 10.1038/s41598-023-37824-4 (2023). 10.1038/s41598-023-37824-4 PMC10333343 37429876

[4] Sundar Ganesh, C. S., Kumar, C., Premkumar, M. & Derebew, B. Enhancing photovoltaic parameter estimation: integration of non-linear hunting and reinforcement learning strategies with golden Jackal optimizer. *Sci. Rep.* **14** (1). 10.1038/s41598-024-52670-8 (2024). 10.1038/s41598-024-52670-8 PMC10837193 38307945

[5] Kullampalayam Murugaiyan, N., Chandrasekaran, K., Manoharan, P. & Derebew, B. Leveraging opposition-based learning for solar photovoltaic model parameter Estimation with exponential distribution optimization algorithm. *Sci. Rep.* **14** (1). 10.1038/s41598-023-50890-y (2024). 10.1038/s41598-023-50890-y PMC10767030 38177405

[6] Qais, M. H., Hasanien, H. M. & Alghuwainem, S. Parameters extraction of three-diode photovoltaic model using computation and Harris Hawks optimization. *Energy* **195**10.1016/j.energy.2020.117040 (2020).

[7] Gnetchejo, P. J., Ndjakomo Essiane, S., Dadjé, A. & Ele, P. A combination of Newton-Raphson method and heuristics algorithms for parameter Estimation in photovoltaic modules. *Heliyon* **7** (4), e06673. 10.1016/J.HELIYON.2021.E06673 (2021). 10.1016/j.heliyon.2021.e06673 PMC8045010 33869869

[8] Kumari, P. A. & Geethanjali, P. Parameter Estimation for photovoltaic system under normal and partial shading conditions: A survey. *Renew. Sustain. Energy Rev.* **84**10.1016/j.rser.2017.10.051 (2018).

[9] Lun, S. et al. An explicit approximate I-V characteristic model of a solar cell based on Padé approximants. *Sol Energy*. **92**10.1016/j.solener.2013.02.021 (2013).

[10] Lo Brano, V. & Ciulla, G. An efficient analytical approach for obtaining a five parameters model of photovoltaic modules using only reference data. *Appl. Energy*. **111**10.1016/j.apenergy.2013.06.046 (2013).

[11] Lun, S. X. et al. A new explicit i-v model of a solar cell based on taylor’s series expansion. *Sol Energy*. **94**10.1016/j.solener.2013.04.013 (2013).

[12] Jain, A. & Kapoor, A. Exact analytical solutions of the parameters of real solar cells using Lambert W-function. *Sol Energy Mater. Sol Cells*. **81** (2), 269–277. 10.1016/j.solmat.2003.11.018 (2004).

[13] Petrone, G. & Spagnuolo, G. Parameters identification of the single-diode model for amorphous photovoltaic panels, in *5th International Conference on Clean Electrical Power: Renewable Energy Resources Impact, ICCEP*, 2015, 2015, (2015). 10.1109/ICCEP.2015.7177608

[14] Rizk, M., Rizk-Allah & El-Fergany, A. A. Emended heap-based optimizer for characterizing performance of industrial solar generating units using triple-diode model. *Energy* **237**, 121561 (2021).

[15] Appelbaum, J. & Peled, A. Parameters extraction of solar cells - A comparative examination of three methods. *Sol Energy Mater. Sol Cells*. **122**10.1016/j.solmat.2013.11.011 (2014).

[16] Tossa, A. K., Soro, Y. M., Azoumah, Y. & Yamegueu, D. A new approach to estimate the performance and energy productivity of photovoltaic modules in real operating conditions. *Sol Energy*. **110**. 10.1016/j.solener.2014.09.043 (2014).

[17] Khanna, V., Das, B. K., Bisht, D., Vandana & Singh, P. K. A three diode model for industrial solar cells and Estimation of solar cell parameters using PSO algorithm. *Renew. Energy*. **78**, 105–113. 10.1016/j.renene.2014.12.072 (2015).

[18] Liang, J. et al. Parameters Estimation of solar photovoltaic models via a self-adaptive ensemble-based differential evolution. *Sol Energy*. **207**, 336–346. 10.1016/j.solener.2020.06.100 (2020).

[19] Jiao, S. et al. Orthogonally adapted Harris Hawks optimization for parameter Estimation of photovoltaic models. *Energy* **203**, 117804. 10.1016/j.energy.2020.117804 (2020).

[20] Yu, K. et al. A performance-guided JAYA algorithm for parameters identification of photovoltaic cell and module. *Appl. Energy*. **237**, 241–257. 10.1016/j.apenergy.2019.01.008 (2019).

[21] Soliman, M. A., Hasanien, H. M. & Alkuhayli, A. Marine predators algorithm for parameters identification of Triple-Diode photovoltaic models. *IEEE Access.* **8**, 155832–155842. 10.1109/ACCESS.2020.3019244 (2020).

[22] Kumar, C., Raj, T. D., Premkumar, M. & Raj, T. D. A new stochastic slime mould optimization algorithm for the Estimation of solar photovoltaic cell parameters. *Optik (Stuttg)*. **223**, 165277. 10.1016/j.ijleo.2020.165277 (2020).

[23] Mahmood, B. S., Hussein, N. K., Aljohani, M. & Qaraad, M. A modified gradient search rule based on the Quasi-Newton method and a new local search technique to improve the gradient-Based algorithm: solar photovoltaic parameter extraction. *Mathematics* **11** (19). 10.3390/math11194200 (2023).

[24] Alsaggaf, W., Gafar, M., Sarhan, S., Shaheen, A. M. & Ginidi, A. R. Chemical-Inspired material generation algorithm (MGA) of Single- and Double-Diode model parameter determination for Multi-Crystalline silicon solar cells. *Appl. Sci.* **14** (18), 8549. 10.3390/app14188549 (2024).

[25] Chaib, L., Tadj, M., Choucha, A., El-Rifaie, A. M. & Shaheen, A. M. Hybrid Brown-Bear and Hippopotamus algorithms with fractional order Chaos maps for precise solar PV model parameter Estimation. *Processes* **12** (12), 2718. 10.3390/pr12122718 (2024).

[26] Premkumar, M., Babu, T. S., Umashankar, S. & Sowmya, R. A new metaphor-less algorithms for the photovoltaic cell parameter Estimation. *Optik (Stuttg)*. **208**, 164559. 10.1016/j.ijleo.2020.164559 (2020).

[27] Sheng, H. et al. Parameters Extraction of Photovoltaic Models Using an Improved Moth-Flame Optimization, *Energies* **12**(18), 3527. 10.3390/EN12183527 (2019).

[28] Hasanien, H. M. Shuffled frog leaping algorithm for photovoltaic model identification. *IEEE Trans. Sustain. Energy*. **6** (2), 509–515. 10.1109/TSTE.2015.2389858 (2015).

[29] Yu, K., Liang, J. J., Qu, B. Y., Chen, X. & Wang, H. Parameters identification of photovoltaic models using an improved JAYA optimization algorithm. *Energy Convers. Manag*. **150**, 742–753. 10.1016/j.enconman.2017.08.063 (2017).

[30] Liao, Z., Chen, Z. & Li, S. Parameters extraction of photovoltaic models using Triple-Phase Teaching-Learning-Based optimization. *IEEE Access.* **8**, 69937–69952. 10.1109/ACCESS.2020.2984728 (2020).

[31] Oliva, D., Abd El, M., Aziz & Ella Hassanien, A. Parameter Estimation of photovoltaic cells using an improved chaotic Whale optimization algorithm. *Appl. Energy*. **200**10.1016/j.apenergy.2017.05.029 (2017).

[32] Elshahed, M. et al. An Innovative Hunter-Prey-Based Optimization for Electrically Based Single-, Double-, and Triple-Diode Models of Solar Photovoltaic Systems. *Mathematics* **10**(23), 4625. 10.3390/math10234625 (2022).

[33] Ginidi, A. R., Shaheen, A. M., El-Sehiemy, R. A., Hasanien, H. M. & Al-Durra, A. Estimation of electrical parameters of photovoltaic panels using heap-based algorithm. *IET Renew. Power Gener.* **16**(11), 2292–2312. 10.1049/RPG2.12523 (2022).

[34] Duman, S. et al. A powerful meta-heuristic search algorithm for solving global optimization and real-world solar photovoltaic parameter Estimation problems. *Eng. Appl. Artif. Intell.* **111**10.1016/j.engappai.2022.104763 (2022).

[35] Liu, Y. et al. Boosting slime mould algorithm for parameter identification of photovoltaic models. *Energy* **234**10.1016/j.energy.2021.121164 (2021).

[36] Abdel-Basset, M., Mohamed, R., El-Fergany, A., Abouhawwash, M. & Askar, S. S. Parameters identification of PV Triple-Diode model using improved generalized normal distribution algorithm. *Mathematics* **9** (9), 995. 10.3390/math9090995 (2021).

[37] Rawat, N. et al. A new grey Wolf optimization-based parameter Estimation technique of solar photovoltaic. *Sustain. Energy Technol. Assessments*. **57**10.1016/j.seta.2023.103240 (2023).

[38] Qaraad, M. et al. Photovoltaic parameter Estimation using improved moth flame algorithms with local escape operators. *Comput. Electr. Eng.* **106**10.1016/j.compeleceng.2023.108603 (2023).

[39] Su, H. et al. RIME: A physics-based optimization. *Neurocomputing* **532**10.1016/j.neucom.2023.02.010 (2023).

[40] Gu, G., Lou, J. & Wan, H. A multi-strategy improved rime optimization algorithm for three-dimensional USV path planning and global optimization. *Sci. Rep.* **14**, 12603. 10.1038/s41598-024-63188-4 (2024). 38824256 10.1038/s41598-024-63188-4 PMC11144218

[41] Zhai, S., Zhao, X., Zu, G., Lu, L. & Cheng, C. An algorithm for lane detection based on RIME optimization and optimal threshold. *Sci. Rep.* **14**, 27244. 10.1038/s41598-024-76837-5 (2024). 39516249 10.1038/s41598-024-76837-5 PMC11549429

[42] Ma, Z. & Zhang, Y. A study on rolling bearing fault diagnosis using RIME-VMD. *Sci. Rep.* **15**, 4712. 10.1038/s41598-025-89161-3 (2025). 39922891 10.1038/s41598-025-89161-3 PMC11807157

[43] Zhong, R., Yu, J., Zhang, C. & Munetomo, M. SRIME: a strengthened RIME with Latin hypercube sampling and embedded distance-based selection for engineering optimization problems. *Neural Comput. Appl.* **36** (12). 10.1007/s00521-024-09424-4 (2024).

[44] Alam, M. M. et al. AI-based efficiency analysis technique for photovoltaic renewable energy system. *Phys. Scr.* **98** (12), 126006. 10.1088/1402-4896/ad0bb4 (2023).

[45] Al-Dhaifallah, M., Alaas, Z., Rezvani, A., Nguyen Le, B. & Samad, S. RETRACTED: Optimal day-ahead economic/emission scheduling of renewable energy resources based microgrid considering demand side management, *J. Build. Eng.* **76**, 107070. 10.1016/J.JOBE.2023.107070 (2023).

[46] Ortiz-Conde, A., Lugo-Muñoz, D. & García-Sánchez, F. J. An explicit multiexponential model as an alternative to traditional solar cell models with series and shunt resistances. *IEEE J. Photovoltaics*. **2** (3), 261–268. 10.1109/JPHOTOV.2012.2190265 (2012).

[47] Qais, M. H., Hasanien, H. M. & Alghuwainem, S. Identification of electrical parameters for three-diode photovoltaic model using analytical and sunflower optimization algorithm. *Appl. Energy*. **250**, 109–117. 10.1016/j.apenergy.2019.05.013 (2019).

[48] Moustafa, G., Alnami, H., Ginidi, A. R. & Shaheen, A. M. An improved Kepler optimization algorithm for module parameter identification supporting PV power Estimation. *Heliyon* **10** (21), e39902. 10.1016/j.heliyon.2024.e39902 (2024). 39553594 10.1016/j.heliyon.2024.e39902 PMC11566844

[49] Fossum, J. G. & Lindholm, F. A. Theory of Grain-Boundary and intragrain recombination currents in polysilicon p-n-Junction solar cells. *IEEE Trans. Electron. Devices*. **27** (4), 692–700. 10.1109/T-ED.1980.19924 (1980).

[50] Koohi-Kamali, S., Rahim, N. A., Mokhlis, H. & Tyagi, V. V. Photovoltaic electricity generator dynamic modeling methods for smart grid applications: A review. *Renew. Sustain. Energy Rev.* **57**, 131–172. 10.1016/j.rser.2015.12.137 (2016).

[51] Chin, V. J. & Salam, Z. Coyote optimization algorithm for the parameter extraction of photovoltaic cells. *Sol Energy*. **194**, 656–670. 10.1016/j.solener.2019.10.093 (2019).

[52] Zhu, W., Fang, L., Ye, X., Medani, M. & Escorcia-Gutierrez, J. Brain tumor image segmentation with boosted RIME optimization. *Comput. Biol. Med.* **166**, 107551. 10.1016/J.COMPBIOMED.2023.107551 (2023). 10.1016/j.compbiomed.2023.107551 37832284

[53] Li, Y. et al. CDRIME-MTIS: An enhanced rime optimization-driven multi-threshold segmentation for COVID-19 X-ray images, *Comput. Biol. Med.* **169**, 107838. 10.1016/J.COMPBIOMED.2023.107838 (2024). 10.1016/j.compbiomed.2023.107838 38171259

[54] Shaheen, A. M., El-Sehiemy, R. A. & Farrag, S. M. Optimal reactive power dispatch using Backtracking search algorithm. *Aust J. Electr. Electron. Eng.* **13** (3). 10.1080/1448837X.2017.1325134 (2016).

[55] Hakmi, S. H., Alnami, H., Moustafa, G., Ginidi, A. R. & Shaheen, A. M. Modified Rime-Ice growth optimizer with polynomial differential learning operator for Single- and Double-Diode PV parameter Estimation problem. *Electronics* **13** (9), 1611. 10.3390/electronics13091611 (2024).

[56] Zhao, W. et al. Quadratic interpolation optimization (QIO): A new optimization algorithm based on generalized quadratic interpolation and its applications to real-world engineering problems. *Comput. Methods Appl. Mech. Eng.* **417**, 116446. 10.1016/J.CMA.2023.116446 (2023).

[57] Bansal, A. & Jain, A. Comparison of meta-heuristic with evolutionary and local search methods for feature selection. *Stud. Comput. Intell.* **916** (2021).

[58] Blot, A., Kessaci, M. É. & Jourdan, L. Survey and unification of local search techniques in metaheuristics for multi-objective combinatorial optimisation. *J. Heuristics*. **24** (6). 10.1007/s10732-018-9381-1 (2018).

[59] Xiong, Y., Zou, Z. & Cheng, J. Cuckoo search algorithm based on cloud model and its application. *Sci. Rep.* **13** (1). 10.1038/s41598-023-37326-3 (2023). 10.1038/s41598-023-37326-3 PMC10284865 37344537

[60] Zangmo, R. et al. Optimal placement of renewable distributed generators and electric vehicles using multi-population evolution whale optimization algorithm, *Sci. Rep.* **14**, 28447. 10.1038/s41598-024-80076-z (2024). 10.1038/s41598-024-80076-z PMC11574299 39558060

[61] Gafar, M., Sarhan, S., Ginidi, A. R. & Shaheen, A. M. An improved Bio-Inspired material generation algorithm for engineering optimization problems including PV source penetration in distribution systems. *Appl. Sci.* **15** (2), 603. 10.3390/app15020603 (2025).

[62] Tong, N. T. & Pora, W. A parameter extraction technique exploiting intrinsic properties of solar cells. *Appl. Energy*. **176**, 104–115. 10.1016/j.apenergy.2016.05.064 (2016).

[63] Easwarakhanthan, T., Bottin, J., Bouhouch, I. & Boutrit, C. Nonlinear minimization algorithm for determining the solar cell parameters with microcomputers. *Int. J. Sol Energy*. **4** (1), 1–12. 10.1080/01425918608909835 (1986).

[64] El-Sehiemy, R., Elsayed, A., Shaheen, A., Elattar, E. & Ginidi, A. Scheduling of generation stations, OLTC substation Transformers and VAR sources for sustainable power system operation using SNS optimizer. *Sustainability* **13** (21), 11947. 10.3390/su132111947 (2021).

[65] Akbari, E., Ghasemi, M., Gil, M., Rahimnejad, A. & Gadsden, S. A. Optimal power flow via teaching-learning-studying-based optimization algorithm. 1971331. 10.1080/15325008.2021.1971331 (2021).

[66] Sarhan, S., El-Sehiemy, R. A., Shaheen, A. M. & Gafar, M. TLBO merged with studying effect for economic environmental energy management in high voltage AC networks hybridized with Multi-Terminal DC lines. *Appl. Soft Comput.* **143**, 110426. 10.1016/J.ASOC.2023.110426 (2023).

[67] Sarhan, S., Shaheen, A., El-Sehiemy, R., Gafar, M., Multi-Objective, A. & Teaching-Learning Studying-Based algorithm for Large-Scale dispatching of combined electrical power and heat energies. *Math* **10** (13), 2278. 10.3390/math10132278 (2022).

[68] Xie, L. et al. Tuna Swarm Optimization: A Novel Swarm-Based Metaheuristic Algorithm for Global Optimization, *Comput. Intell. Neurosci.*. 10.1155/2021/9210050 (2021). 10.1155/2021/9210050 PMC8550856 34721567

[69] Selem, S. I., El-Fergany, A. A. & Hasanien, H. M. Artificial electric field algorithm to extract nine parameters of triple-diode photovoltaic model. *Int. J. Energy Res.*10.1002/er.5756 (2021).

[70] Abdollahzadeh, B., Gharehchopogh, F. S. & Mirjalili, S. African vultures optimization algorithm: A new nature-inspired metaheuristic algorithm for global optimization problems. *Comput. Ind. Eng.* **158**. 10.1016/j.cie.2021.107408 (2021).

[71] Shaheen, A., El-Sehiemy, R., El-Fergany, A. & Ginidi, A. Representations of solar photovoltaic triple-diode models using artificial hummingbird optimizer. *Energy Sources Part. Recover Util. Environ. Eff.* **44** (4), 8787–8810. 10.1080/15567036.2022.2125126 (2022).

[72] Shaheen, A. M., Elsayed, A. M., Ginidi, A. R., El-Sehiemy, R. A. & Elattar, E. Enhanced social network search algorithm with powerful exploitation strategy for PV parameters Estimation. *Energy Sci. Eng.* **10** (4), 1398–1417. 10.1002/ese3.1109 (2022).

[73] Kang, T., Yao, J., Jin, M., Yang, S. & Duong, T. A novel improved cuckoo search algorithm for parameter Estimation of photovoltaic (PV) models. *Energies* **11** (5). 10.3390/en11051060 (2018).

[74] Chen, X. & Yu, K. Hybridizing cuckoo search algorithm with biogeography-based optimization for estimating photovoltaic model parameters. *Sol Energy*. **180**, 192–206. 10.1016/j.solener.2019.01.025 (2019).

[75] El-Dabah, M. A., El-Sehiemy, R. A., Becherif, M. & Ebrahim, M. A. Parameter estimation of triple diode photovoltaic model using an artificial ecosystem-based optimizer, *Int. Trans. Electr. Energy Syst.* e13043. 10.1002/2050-7038.13043 (2021).

[76] Ben Aribia, H. et al. Growth optimizer for parameter identification of solar photovoltaic cells and modules. *Sustainability* **15** (10), 7896. 10.3390/su15107896 (2023).

[77] Oliva, D., Cuevas, E. & Pajares, G. Parameter identification of solar cells using artificial bee colony optimization. *Energy* **72**, 93–102. 10.1016/j.energy.2014.05.011 (2014).

[78] Chen, X., Xu, B., Mei, C., Ding, Y. & Li, K. Teaching–learning–based artificial bee colony for solar photovoltaic parameter Estimation. *Appl. Energy*. **212**, 1578–1588. 10.1016/j.apenergy.2017.12.115 (2018).

[79] Xu, S. & Wang, Y. Parameter estimation of photovoltaic modules using a hybrid flower pollination algorithm. *Energy Convers. Manag.* **144**, 53–68. 10.1016/J.ENCONMAN.2017.04.042 (2017).

[80] Chen, H. et al. An opposition-based sine cosine approach with local search for parameter estimation of photovoltaic models, *Energy Convers. Manag.* **195**, 927–942. 10.1016/J.ENCONMAN.2019.05.057 (2019).

[81] Rao, R. V., Savsani, V. J. & Vakharia, D. P. Teaching-Learning-Based optimization: an optimization method for continuous non-linear large scale problems. *Inf. Sci. (Ny)*. **183** (1). 10.1016/j.ins.2011.08.006 (2012).

[82] Guo, L., Meng, Z., Sun, Y. & Wang, L. Parameter identification and sensitivity analysis of solar cell models with Cat swarm optimization algorithm. *Energy Convers. Manag*. **108**, 520–528. 10.1016/j.enconman.2015.11.041 (2016).

[83] Liang, J. J., Qin, A. K., Suganthan, P. N. & Baskar, S. Comprehensive learning particle swarm optimizer for global optimization of multimodal functions, *IEEE Trans. Evol. Comput.* **10**(3), 281–295. 10.1109/TEVC.2005.857610 (2006).

[84] Chen, X., Yu, K., Du, W., Zhao, W. & Liu, G. Parameters identification of solar cell models using generalized oppositional teaching learning based optimization. *Energy* **99**, 170–180. 10.1016/j.energy.2016.01.052 (2016).

