# Supplementary material for: Enhancement of rime algorithm using quadratic interpolation learning for parameters identification of photovoltaic models
Source: Sci Rep. 2025 Jul 1;15:21166. doi: 10.1038/s41598-025-04589-x (PMC12216348; doi:10.1038/s41598-025-04589-x)
Supplement: Supplementary file 1 — Supplementary Material 1 [file 41598_2025_4589_MOESM1_ESM.pdf]

Appendix

The following algorithm (Algorithm. 1) displays the MATLAB code for the proposed IRMO with QIL for solving the sphere function.

Algorithm. 1 Rime Algorithm with Quadratic Interpolation Learning for solving Sphere function

```
% Initialize position
dim = 30; % Number of dimensions
Best_rime=zeros(1,dim);
Best_rime_rate=inf; %change this to -inf for maximization problems
Lb= -100; % Lower bound of search space
Ub= 100; % Upper bound of search space
N= 50; % Population size
Rime_rates=zeros(1,N); %Initialize the fitness value
newRime_rates=zeros(1,N);
W = 5;

% Initialize population
    Rimepop = Lb + rand(N, dim).* (Ub-Lb); % Random initial positions (%Equation 10)
    Rime_rates = SphereFunction(Rimepop); % Evaluate the fitness

    % Identify the best bird
    [Best_rime_rate, bestIndex] = min(Rime_rates);
    Best_rime = Rimepop (bestIndex, :);

% Loop =========
for iter=1:MaxIt
    RimeFactor = (rand-0.5)*2*cos((pi*iter/(MaxIt/10)))*(1-round(iter*W/MaxIt)/W); (%Equation 10)
    EN =sqrt(iter/MaxIt);
    newRimepop = Rimepop;
    normalized_rime_rates=normr(Rime_rates); (%Equation 16)
    for i=1:SS
        if rand >0.5
        for j=1:dim
            %Soft-rime search strategy
            r1=rand();
            if r1< EN
                newRimepop(i,j)=Best_rime(1,j)+RimeFactor*((Ub(j)-Lb(j))*rand+Lb(j)); (%Equation 11)
            end
            %Hard-rime puncture mechanism
            r2=rand();
            if r2<normalized_rime_rates(i)
                newRimepop(i,j)=Best_rime(1,j); (%Equation 15)
            end
        end
```

```
        else

% Quadratic Interpolation
            K1=randi(SS);  K2=randi(SS);
            f1=Rime_rates(i); f2=Rime_rates(K1); f3=Rime_rates(K2);
            for j=1:dim
                x1=Rimepop(i,j);  x2=Rimepop(K1,j); x3=Rimepop(K2,j);
                % Eq.(25)
                newRimepop(i,j)=GQI(x1,x2,x3,f1,f2,f3,Lb(j),Ub(j));
            end
        end
    end

for ii=1:SS
        %Boundary absorption
        Flag4ub=newRimepop(ii,:)>Ub;
        Flag4lb=newRimepop(ii,:)<Lb;
        newRimepop(ii,:)=(newRimepop(ii,:).*(~(Flag4ub+Flag4lb)))+Ub.*Flag4ub+Lb.*Flag4lb;
        newRime_rates(1,ii)= SphereFunction(newRimepop(ii)); % Evaluate the fitness
        %Positive greedy selection mechanism (%Equation 17)
        if newRime_rates(1,ii)<Rime_rates(1,ii)
            Rime_rates(1,ii) = newRime_rates(1,ii);
            Rimepop(ii,:) = newRimepop(ii,:);
            if newRime_rates(1,ii)< Best_rime_rate
               Best_rime_rate=Rime_rates(1,ii);
               Best_rime=Rimepop(ii,:);
            end
        end
    end

% Final result
 fprintf('Optimal solution found:\n');
 disp(Best_rime);
 fprintf('Minimum fitness value: %.6f\n', Best_rime_rate);
 end % End of loop

% Sphere function definition (Equation 10)
function f = SphereFunction(X)
    f = sum(X.^2, 2);
end

function L=GQI(a,b,c,fa,fb,fc,low,up) % Generalized Quadratic Interpolation (GQI)
fabc=[fa fb fc];
[fijk,ind]=sort(fabc);
fi=fijk(1);fj=fijk(2);fk=fijk(3);
dim=length(a);
ai=ind(1); bi=ind(2);ci=ind(3);
```

```
L=zeros(1,dim);
for i=1:dim
    x=[a(i) b(i) c(i)];
    xi=x(ai); xj=x(bi); xk=x(ci);
    if (xk>=xi && xi>=xj) || (xj>=xi && xi>=xk)
        L(i)=Interpolation(xi,xj,xk,fi,fj,fk,low(i),up(i));
    elseif (xk>=xj && xj>=xi)
        I=Interpolation(xi,xj,xk,fi,fj,fk,low(i),up(i));
        if  I<xj
            L(i)=I;
        else
            L(i)=Interpolation(xi,xj,3*xi-2*xj,fi,fj,fk,low(i),up(i));
        end
    elseif (xi>=xj && xj>=xk)
        I=Interpolation(xi,xj,xk,fi,fj,fk,low(i),up(i));
        if  I>xj
            L(i)=I;
        else
            L(i)=Interpolation(xi,xj,3*xi-2*xj,fi,fj,fk,low(i),up(i));
        end
    elseif (xj>=xk && xk>=xi)
        L(i)=Interpolation(xi,2*xi-xk,xk,fi,fj,fk,low(i),up(i));
    elseif (xi>=xk && xk>=xj)
        L(i)=Interpolation(xi,2*xi-xk,xk,fi,fj,fk,low(i),up(i));
    end
end

function L_xmin=Interpolation(xi,xj,xk,fi,fj,fk,l,u) % Quadratic interpolation
  a=(xj^2-xk^2)*fi+(xk^2-xi^2)*fj+(xi^2-xj^2)*fk;
  b=2*((xj-xk)*fi+(xk-xi)*fj+(xi-xj)*fk);
  L_xmin=a/(b+eps);
  if isnan(L_xmin) || isinf(L_xmin) || L_xmin>u || L_xmin<l
      L_xmin=(rand*(u-l)+l);
  end
```
